# Antioxidant and Antidiabetic Activity of the Bulb Extract of *Crinum amoenum* Roxb. ex Ker Gawl: An Integrated In Vitro, In Vivo and In Silico Approach

**DOI:** 10.3390/antiox15081004

**Published:** 2026-08-13

**Authors:** Prabhat Kumar Jha, Kalsoom Khan, Asad Abbas, Ralf Weiskirchen, Bibek Kumar Kohar, Namrata Bhattarai, Ram Kishor Yadav, Biswash Sapkota, Abdul Malik, Sushil Panta, Bipindra Pandey

**Affiliations:** 1 Department of Pharmacy, Madan Bhandari Academy of Health Sciences, Hetauda 44100, Nepal; prabhat.jha@mbahs.edu.np (P.K.J.); bibek.koharp3@mbahs.edu.np (B.K.K.); biswash.sapkota@mbahs.edu.np (B.S.); 2 School of Health and Allied Sciences, Pokhara University, Pokhara 33700, Nepal; namrata.bhattarai_mpharm2023@student.pu.edu.np (N.B.); ramkishor.yadav@student.pu.edu.np (R.K.Y.); sushil.panta@pu.edu.np (S.P.); 3 Department of Health and Biological Sciences, Abasyn University, Peshawar 25000, Pakistan; kalsoom20547@abasyn.edu.pk; 4 Department of Human Nutrition, Faculty of Food Science and Nutrition, Bahauddin Zakariya University, Multan 60800, Pakistan; asadabbaskhichi@gmail.com; 5 Institute of Molecular Pathobiochemistry, Experimental Gene Therapy and Clinical Chemistry (IFMPEGKC), RWTH University Hospital Aachen, D-52074 Aachen, Germany; 6 Department of Pharmaceutics, College of Pharmacy, King Saud University, P.O. Box 2457, Riyadh 11451, Saudi Arabia; amoinuddin@ksu.edu.sa

**Keywords:** Amaryllidaceae alkaloids, α-amylase inhibition, postprandial hyperglycemia, molecular dynamics simulation, in silico pharmacokinetics, oral glucose tolerance, ADMET prediction

## Abstract

*Crinum amoenum* Roxb. ex Ker Gawl. (*C. amoenum*) is traditionally used in Nepal, but its antidiabetic potential remains insufficiently validated. This study characterized the 80% (*v*/*v*) ethanol bulb extract of *C. amoenum* and evaluated its antioxidant, α-amylase inhibitory, hypoglycemic, molecular docking, molecular dynamics (MD) simulation, and ADMET profiles. The ethanolic bulb extract was evaluated through phytochemical screening, thin-layer chromatography, total phenolic content (TPC), total flavonoid content (TFC) estimation, LC-MS analysis, antioxidant assays, α-amylase inhibition assays, acute toxicity testing, and hypoglycemic/oral glucose tolerance test (OGTT) studies in Wistar rats. Lycorine, pratorinine, and palmatine were docked against α-amylase (PDB ID: 5U3A) and GLP-1R (PDB ID: 6GB1), followed by 200 ns MD simulations and ADMET prediction. Qualitative analysis of the extract was positive for flavonoids, phenolic compounds, tannins, saponins, alkaloids, and carbohydrates, and a positive Salkowski reaction indicated the presence of constituents occurring in glycosidic form. The TPC was 173.38 ± 1.22 mg GAE/g, and the TFC was 314.58 ± 0.09 mg QE/g. LC-MS tentatively annotated lycorine, pratorinine, and palmatine based on retention time, m/z values in positive-mode electrospray ionization, and comparison with previously reported spectral information. The extract showed DPPH scavenging activity (IC_50_: 90.98 μg/mL) and strong α-amylase inhibition (IC_50_: 49.31 μg/mL) compared with acarbose (IC_50_: 51.51 μg/mL). No deaths were observed following oral administration at 5000 mg/kg to rats. The 500 mg/kg dose produced the greatest hypoglycemic response, reducing blood glucose by 35.78% at 120 min in non-diabetic Wistar rats and by 19.21% at 60 min in glucose-loaded rats. Among all the compounds tested, pratorinine demonstrated the highest docking affinity with α-amylase (−8.2 kcal/mol; ASP300) and GLP-1R (−7.0 kcal/mol; GLY132). MD simulation supported greater stability of the α-amylase complex, and ADMET prediction identified pratorinine as a comparatively favorable predicted lead candidate (LD_50_: 1000 mg/kg; Class 4 toxicity category). Pratorinine was identified as the most promising in silico antidiabetic candidate from the *C. amoenum* extract, primarily due to stable α-amylase interactions and favorable ADMET properties.

## 1. Introduction

Plant-derived remedies remain integral to traditional healthcare systems and continue to be an active area of scientific investigation today, including the development of frameworks that protect and sustainably manage plant-derived intellectual property [1]. In many countries, they play a crucial role in healthcare systems and are often referred to as complementary and alternative medicine. According to the World Health Organization, traditional medicine encompasses a variety of health practices, approaches, knowledge, and beliefs. These include phytomedicines, animal products, and mineral drugs, as well as spiritual, manual, and exercise-based interventions, either individually or in combination, to promote health or prevent illness [2]. Additionally, according to the World Health Organization, 60–85% of people worldwide rely on complementary and traditional medicine for their basic medical needs [3].

Nepal is home to a variety of ethnic communities, each with distinct traditional medicinal practices. Traditional Nepalese medicine comprises various ancient therapeutic traditions, including Ayurveda, traditional Tibetan medicine, and indigenous folk practices [4]. With the growing recognition of naturopathy and holistic medicine, Nepal’s traditional medical system offers significant insights and therapeutic options. It embodies a distinctive amalgamation of traditional knowledge, cultural diversity, and the therapeutic potential of nature, promoting a holistic approach to well-being [5]. In Nepal, a variety of medical conditions are commonly treated with traditional remedies. *C. amoenum* Roxb. ex Ker Gawl. (*C. amoenum*) belongs to the Amaryllidaceae family and is also known as the Himalayan crinum lily. It is characterized by slender, spreading petals, long green floral tubes, prominent reddish stamens, and leaves measuring 10–12 cm in length and 2.5–4 cm in width. The plant is popularly known as hadelasun in the western region of Nepal, particularly in Gandaki Province, while in eastern Nepal, it is locally referred to as gorasun. *C. amoenum* is distributed from Nepal through Northeast India to Bangladesh.

The bulb of *C. amoenum* is used by the Rajbanshi community in the southern region of Nepal to treat dysmenorrhoea [6]. Similarly, the Rai community in the Bhojpur District of eastern Nepal utilizes the rhizome to prepare a paste used to treat cholera and dysentery [7]. Local practitioners in the Kaski District report that the bulb of this herb is administered to cattle to treat gastrointestinal (GI) problems, especially abdominal distension. However, this ethnoveterinary use has not yet been scientifically documented and remains based on oral knowledge from traditional healers in Pokhara and Kaski. Various studies on *C. amoenum* have demonstrated its abundance of phytochemicals, including terpenoids, alkaloids, flavonoids, saponins, and cardiac glycosides [8,9]. However, there are still few systematic attempts in the literature that validate its ethnopharmacological uses or isolate and quantify its bioactive components.

The genus *Crinum* is a recognized source of Amaryllidaceae isoquinoline alkaloids, and several species accumulate lycorine-type, phenanthridone-type, and protoberberine bases, together with phenolic acids and flavonoids [9]. Species within this genus have shown hypoglycemic effects in addition to the ability to block α-amylase and α-glucosidase in rodent models [10,11]. Alkaloid-rich fractions of *Crinum* species inhibit α-amylase and α-glucosidase in vitro, and hydroalcoholic extracts of *C. abyssinicum* reduce fasting glucose in mice [11]. Comparable data for *C. amoenum* are limited to preliminary antioxidant and antibacterial screening [8,9], and no study has combined chemical annotation with enzymatic, in vivo, and computational evaluation for this species.

Pancreatic α-amylase catalyzes the first step in the hydrolysis of dietary starch, and its inhibition delays the release of glucose into the circulation, which is the accepted basis of the postprandial action of acarbose [12,13]. A unique, incretin-driven approach of glycemic control is to target the glucagon-like peptide-1 receptor (GLP-1R) [13]. These two targets were therefore selected to test whether the constituents of the bulb extract act mainly through inhibition of starch digestion or through an additional receptor-mediated route.

Three alkaloids were taken for computational study in the present work: lycorine, pratorinine, and palmatine. Lycorine is the parent compound of the lycorine-type Amaryllidaceae alkaloids, with a pyrrolo[de]phenanthridine skeleton bearing a methylenedioxy group and two hydroxyl functions, and it has documented cytotoxic, antiviral, antimalarial, and acetylcholinesterase inhibitory activities [14,15,16]. Pratorinine belongs to the oxophenanthridine (phenanthridone) subgroup of the same alkaloid family, and its planar and highly conjugated ring system, together with one phenolic hydroxyl and one methoxy substituent, favors hydrogen bonding and π-stacking within enzyme-binding pockets [13]. Palmatine is a quaternary protoberberine alkaloid that has been reported to reduce blood glucose in animal models and to inhibit carbohydrate hydrolyzing enzymes [13]. These three compounds were selected for docking, molecular dynamics simulation, and ADMET prediction because they were the constituents tentatively annotated in the bulb extract in the present study and because their reported pharmacology is consistent with the α-amylase inhibitory activity recorded here.

Despite its long history of use, little is known about this species’ pharmacology and phytochemistry, and the present investigation was therefore undertaken to screen the 80% ethanol bulb extract of *C. amoenum* for the main classes of secondary metabolites, to quantify its total phenolic and flavonoid contents, to tentatively annotate its major alkaloids by LC-MS, to evaluate its radical-scavenging capacity, reducing power, and α-amylase inhibitory activity in vitro, to assess acute oral toxicity and hypoglycemic activity in Wistar rats, and to examine the interaction of the annotated alkaloids with α-amylase and GLP-1R by molecular docking, 200 ns molecular dynamics simulation, and ADMET prediction. Isolation and quantification of individual alkaloids were outside the scope of this work and are proposed as the next stage of investigation. Moreover, to our knowledge, this is the first integrated study to combine in vitro and in vivo hypoglycemic assessment, molecular docking, MD simulation, and ADMET analysis of the 80% ethanol bulb extract of *C. amoenum*.

## 2. Materials and Methods

### 2.1. Ethics Statement

This study was conducted in accordance with the National Institutes of Health (NIH) guidelines for the use and care of laboratory animals. The study procedure for animal experiments (Protocol Ref. No. 105-079/80; approval date: 9 January 2023) was authorized by the Institutional Review Committee (IRC) of the Pokhara University Research Center (PURC). The ethical approval letter is provided as Supplementary Ethical Documentation. All procedures involving animal subjects complied with NIH standards and guidelines, and every attempt was made to reduce animal suffering.

### 2.2. Plant Material and Chemicals

In October 2022, fresh *C. amoenum* bulbs were collected from Pokhara-31, Kaski, Nepal. A taxonomist at the National Herbarium and Plant Laboratory in Godavari, Lalitpur, Nepal, identified the specimen (Ref. No. 11-079/08). A voucher specimen with Herbarium Sheet Registration Number PUH-2022-41 was deposited in Pokhara University’s Pharmacognosy Laboratory. The IUCN Red List does not list this species as endangered, vulnerable, threatened, or protected.

Ascorbic acid, curcumin, and 2,2-diphenyl-1-picrylhydrazyl (DPPH) were acquired from Sigma-Aldrich (St. Louis, MO, USA). Ethanol, ferric chloride, aluminum chloride, gallic acid, hydrochloric acid, hydrogen peroxide, iodine, potassium ferricyanide, quercetin, and sodium carbonate were among the standard-grade chemicals purchased from Merck (Darmstadt, Germany). The Folin–Ciocalteu reagent and α-amylase were purchased from Sigma-Aldrich. The remaining chemicals and solvents utilized in this investigation were all of analytical reagent grade.

### 2.3. Preparation of C. amoenum Bulb Extract

After being carefully cleaned with distilled water, fresh bulbs were air-dried in the dark for fifteen days. The bulbs were ground into a coarse powder using a grinder once they had dried. Prior to extraction, the powdered material was not sieved. Using the method of Afzal et al. [17], cold maceration was performed with slight modifications. An 80% (*v*/*v*) ethanol solution was selected due to its ability to extract a broad range of phenolic compounds and flavonoids. In order to guarantee sufficient solvent penetration and extraction efficiency, the 1:8 (*w*/*v*) liquid-to-solid ratio described in the method was used. Whatman No. 1 filter paper was used to filter the mixture, and ethanol was removed from the filtrate using a rotary evaporator (Biobase RE-2000, Merck, Darmstadt, Germany) at 40 °C under reduced pressure (120 mbar). After being dried in a vacuum desiccator, the crude extract was stored at 4 °C for up to one month.

### 2.4. Phytochemical Analysis

#### 2.4.1. Qualitative Phytochemical Analysis

##### Phytochemical Screening

Phytochemical screening was performed to identify the major classes of chemical constituents in the extract using colorimetric reactions with various reagents [8,18]. The presence of flavonoids, carbohydrates, glycosides, alkaloids, tannins, saponins, proteins, and steroids in the *C. amoenum* extract was qualitatively tested.

##### Thin-Layer Chromatography Profiling

The thin-layer chromatography (TLC) process was carried out in accordance with our earlier research [19]. A particle-free solution of the 80% ethanol bulb extract of *C. amoenum* (1 mg/mL) was applied using a microcapillary tube (Remediolife, Haryana, India) onto silica gel 60 F254 plates. The glass chamber used to develop the plates was pre-saturated with chloroform, methanol, and water (7:3:0.5, *v*/*v*/*v*), dried in a stream of hot air, and visualized under UV light at 254 and 365 nm. After that, the plates were immersed in DPPH solution (500 µM in methanol) and examined after 10 min. Decolorized zones against the violet background were recorded as radical-scavenging bands. The procedure was used as a qualitative screen only, and no attempt was made to assign structures to the separated bands.

#### 2.4.2. Quantitative Phytochemical Analysis

##### Total Phenolic Content

The Folin–Ciocalteu reagent method was used to spectrophotometrically determine the total phenolic content (TPC) of the *C. amoenum* extract [20]. In short, 6 mL of distilled water was combined with 100 μL of the extract (1000 μg/mL in methanol) and 0.5 mL of 2 N Folin–Ciocalteu reagent for 10 s using a vortex mixer (BJPX-VW, Biobase, Jinan, China). After five minutes, 1.9 mL of distilled water and 1.5 mL of 7.5% sodium carbonate were added, thoroughly mixed, and incubated for two hours in the dark. Gallic acid (12.5–500 μg/mL) was used as the standard reference phenolic compound for the calibration curve and was treated identically to the extract. An Agilent Cary 60 single-beam UV-Vis spectrophotometer (Agilent Technologies, Santa Clara, CA, USA) was used to measure absorbance at 750 nm. Under the same circumstances, 100 μL of methanol was used in place of the extract to provide a control solution. The TPC was expressed as milligrams of gallic acid equivalents (mg GAE) per gram of extract, and all tests were performed in triplicate.

##### Total Flavonoid Content

The total flavonoid content (TFC) of the *C. amoenum* extract was determined using the aluminum chloride (AlCl_3_) method [21]. Equal volumes of AlCl_3_ solution (2% in methanol) and *C. amoenum* extract (100 μg/mL in methanol) were mixed to obtain a final volume of 2 mL. After that, the mixture was incubated for ten minutes at room temperature. Quercetin was used as the reference flavonoid to create a calibration curve. Quercetin concentrations ranged from 50 to 500 μg/mL, and the extract was prepared using the same method. A single-beam UV-Vis spectrophotometer was used to measure absorbance at 415 nm against a methanol blank. The TFC, expressed as milligrams of quercetin equivalents (mg QE) per gram of extract, was computed using the mean absorbance of three replicates for each sample.

### 2.5. Liquid Chromatography–Mass Spectrometry Analysis

The 80% ethanol extract of *C. amoenum* was examined using a liquid chromatography–electrospray ionization mass spectrometry (LC–ESI/MS) system [22]. A Shimadzu HPLC system (LCMS-2020; Shimadzu Corporation, Kyoto, Japan) and a Waters XBridge C18 column (50 × 4.6 mm, 3.5 μm; Waters Corporation, Milford, MA, USA) maintained at 35 °C were used for liquid chromatography. The mobile phases consisted of 0.1% formic acid in water (A) and pure acetonitrile (B), with a flow rate of 1.2 mL/min using the following linear gradient: 0 min, 85/15% A/B; 6 min, 25/75% A/B; and 11–15 min, 85/15% A/B. The injection volume was 5 μL of a particle-free extract solution (1 mg/mL).

Using electrospray ionization in positive mode (ESI+) for metabolite detection and annotation, a single-quadrupole mass analyzer (LCMS-2020) was utilized for mass spectrometric detection, scanning from m/z 0 to 1000. The mass spectrometry source parameters were as follows: ESI capillary voltage, 3.03 kV; cone voltage, 13 V; source temperature, 118 °C; desolvation temperature, 246 °C; desolvation gas flow rate, 500 L/h; and cone gas flow rate, 50 L/h.

Compounds were tentatively annotated by comparing the obtained retention times and m/z values with information published in the literature [22,23] and with entries in the MassBank of Europe (https://massbank.eu/MassBank/; last accessed 11 July 2026) and the National Library of Medicine database, PubChem (https://www.nlm.nih.gov/; last accessed 11 July 2026). Annotation was based on positive-mode electrospray ionisation data only. Because authentic standards and NMR data were not available, all annotations are reported at Metabolomics Standards Initiative confidence level 3 and are described throughout as tentative.

### 2.6. In Vitro Biological Activity

#### 2.6.1. Antioxidant Activities

##### DPPH Radical-Scavenging Assay

With a few minor adjustments, 2 mL of *C. amoenum* extract and 2 mL of 100 µM DPPH solution in ethanol were combined to assess the capacity of the plant extract to scavenge DPPH radicals [19]. After shaking, the mixture was left to stand for half an hour at room temperature in the dark. A UV-Vis spectrophotometer was used to measure absorbance at 517 nm and the DPPH radical-scavenging activity was calculated using the following equation:

$$DPPH\;radical\;scavenging\;rate\;(\%)=\frac{A_{o}-As}{A_{o}}\times 100$$

where A_o_ represents the absorbance of the control, containing all reagents except the test sample, and A_s_ represents the absorbance of the test sample. Ascorbic acid was used as the positive control. The antioxidant activity of each sample was expressed as IC_50_, which indicates the concentration required to scavenge 50% of DPPH radicals.

##### Nitric Oxide Scavenging Activity

By incubating sodium nitroprusside (SNP) with *C. amoenum* extract at doses ranging from 0.1 to 1000 µg/mL at pH 7.2 for 2.5 h at 29 °C, the nitric oxide radical-scavenging activity was evaluated. Nitrite production was assessed by adding Griess reagent, and absorbance was measured at 548 nm using a UV spectrophotometer [24]. The radical-scavenging activity of the extract was calculated using the following equation:

$$Nitric\;oxide\;scavenging\;rate\;(\%)=\frac{A_{o}-As}{A_{o}}\times 100$$

where A_o_ represents the absorbance of the control, containing all reagents except the test sample, and A_s_ represents the absorbance of the test sample. Curcumin was used as the positive control.

##### Hydrogen Peroxide Scavenging Activity

With a few minor modifications, our previously reported approach was used to measure hydrogen peroxide scavenging activity [19]. *C. amoenum* extract (1 mL) at various concentrations (0.1, 1, 10, 100, 250, 500, and 1000 µg/mL) was mixed with 20 mM H_2_O_2_ solution (1 mL) prepared in 0.1 M phosphate buffer (2 mL, pH 7.4) and incubated for 10 min. Absorbance was recorded at 230 nm. The hydrogen peroxide scavenging activity was calculated using the following equation:

$$Hydrogen\;peroxide\;scavenging\;rate\;(\%)=\frac{A_{o}-As}{A_{o}}\times 100$$

where A_o_ represents the absorbance of the control, containing all reagents except the test sample, and A_s_ represents the absorbance of the test sample. Ascorbic acid was used as the positive control.

##### Ferric Reducing Antioxidant Power

As previously mentioned, a modified ferric reducing antioxidant power assay (FRAP) was used to assess the reducing capacity of *C. amoenum* extract [19,25]. Phosphate buffer (0.2 M, pH 6.6) and 1% potassium ferricyanide (2.5 mL) were combined with extracts at different concentrations ranging from 12.5 to 100 µg/mL. The mixture was then incubated at 50 °C for 20 min. After adding 2.5 mL of 10% trichloroacetic acid to stop the reaction, the liquid was centrifuged for 10 min at 6000× *g*. Lastly, 0.5 mL of 0.1% ferric chloride, 2.5 mL of distilled water, and 2.5 mL of the supernatant were combined. A UV-Vis spectrophotometer was used to measure absorbance at 700 nm. Ascorbic acid was used as the positive control.

#### 2.6.2. In Vitro α-Amylase Inhibitory Activity of *C. amoenum*

As previously mentioned, the chromogenic 3,5-dinitrosalicylic acid (DNSA) technique was used to measure the α-amylase inhibitory activity [12]. Porcine pancreatic α-amylase (50 µg/mL) and *C. amoenum* extract (100 µg/mL) were combined and incubated for ten minutes at 37 °C. The substrate was a 1% starch solution, and the control was α-amylase without *C. amoenum* extract. A UV-Vis spectrophotometer was utilized to measure the reducing sugar levels at 540 nm utilizing the DNSA assay, and α-amylase inhibitory activity was calculated using the following equation:

$$Inhibition\;(\%)=\frac{Absorbance\;of\;control-Absorbance\;of\;sample}{Absorbance\;of\;control}\times 100$$

The inhibitory activity was evaluated by calculating percentage inhibition and IC_50_ values based on the concentration–response effect of *C. amoenum* bulb extract on porcine pancreatic α-amylase [26]. For ten minutes, starch (1–5 mg/mL) was incubated with *C. amoenum* bulb extract and porcine pancreatic α-amylase. The DNSA method was then used to measure the amount of residual enzyme activity. The IC_50_ values were derived from plots of percentage inhibition against inhibitor concentration and calculated using logarithmic regression analysis.

### 2.7. In Vivo Activity of C. amoenum Bulb Extract

#### 2.7.1. Experimental Animals, Housing, and Humane Endpoint

The Department of Plant Resources’ Animal House Division in Kathmandu, Nepal, provided 35 healthy Wistar albino rats (n = 35), weighing 200–250 g and aged 8–10 weeks. Of these, thirty male Wistar rats were randomly allocated into five groups (n = 6/group) and used to evaluate hypoglycemic activity in normoglycemic rats and oral glucose tolerance test (OGTT) responses. For the acute toxicity investigation, the remaining five female rats were nulliparous, healthy, and not pregnant.

Animals showing signs of illness, abnormal behavior, injury, pregnancy, or failure to acclimatize were excluded from this study. However, no animals were excluded after group allocation, and all thirty rats included in the hypoglycemic/OGTT experiment were analyzed. The sample size was chosen based on prior comparable pharmacological research and the ethical principle of minimizing animal use. No formal power calculation was carried out.

In the Pokhara University animal research facility, the animals were kept in polypropylene cages with a natural 12 h light/dark cycle, room temperature of 25 ± 3 °C, and ambient humidity. The rats were fed a standard diet and given unlimited access to water for two weeks in compliance with the National Research Council’s recommendations [27].

Before the experiments began, all investigators responsible for treatment administration and/or outcome assessment were blinded where applicable. Animals were randomly allocated to different groups using a computer-generated randomization procedure. Allocation was performed after acclimatization, and each group contained six animals with comparable baseline body weights.

All animal experiment were carried out strictly in compliance with NIH guidelines. All efforts were made to lessen animal suffering in compliance with the National Center for the Replacement, Refinement, and Reduction of Animals in Research (NC3Rs) guidelines, as described in the Animal Research: Reporting of In Vivo Experiments (ARRIVE) guidelines. At the end of this study, all animals were deeply anesthetized by intramuscular injection of ketamine (87 mg/kg) and xylazine (13 mg/kg) [28], followed by euthanasia via cervical dislocation.

#### 2.7.2. Acute Toxicity Study

Five healthy, non-pregnant, nulliparous female Wistar albino rats were chosen at random to evaluate the acute toxicity of the ethanolic extract of *C. amoenum* in compliance with OECD guideline 425. Before the toxicity study, the rats were fasted for 18 h. Animals received sequential administration of extract doses of 2000, 3000, and 5000 mg/kg according to the study design, while control animals received distilled water [29]. Convulsions, writhing reflex, diarrhea, weight loss, lethargy, tremors, paralysis, urination, food and water intake, morbidity, and death were observed continuously for four hours at 30 min intervals, and then daily for two weeks.

#### 2.7.3. In Vivo Determination of Hypoglycemic Activity

The hypoglycemic effect of *C. amoenum* extract was assessed in vivo in Wistar albino rats using a slightly modified protocol described by Reza et al. [30]. Thirty healthy albino rats were randomly divided into five groups (n = 6 rats per group). The control group was given 2% Tween solution orally, while the standard group was given metformin (100 mg/kg, p.o.). The test groups were given 125, 250, and 500 mg/kg of *C. amoenum* ethanolic extract, respectively.

The hypoglycemic activity experiment in normoglycemic rats was conducted first, followed by the OGTT experiment after a one-week washout period to minimize carryover effects. Animals were fasted for 18 h before each glucose measurement experiment. Reuse of the same experimental animals was performed to reduce unnecessary animal use while maintaining ethical standards. All animals were given free access to water and fasted for 18 h before the experiment. Fasting blood glucose levels were measured at the beginning of the trial, and the corresponding treatments were administered orally. After a 1 h interval, all groups received dextrose solution (2 g/kg, p.o.). Following the manufacturer’s instructions, tail vein blood was used to measure blood glucose levels at 0, 30, 60, 120, and 180 min after glucose administration using a calibrated electronic glucometer.

### 2.8. In Silico Studies

#### 2.8.1. Molecular Docking

##### Design of Ligands and Proteins

Using PDB IDs 5U3A and 6GB1, respectively, the three-dimensional crystal structures of the antidiabetic targets α-amylase and the GLP-1 receptor (GLP-1R) domain were obtained from the Protein Data Bank (PDB), and high-resolution structures were chosen. The protein structures were prepared by removing co-crystallized ligands, non-essential ions, and water molecules, followed by the addition of missing hydrogen atoms and assignment of appropriate protonation states at physiological pH (7.4). The structures were energy-minimized using a suitable force field to remove steric conflicts and optimize geometry after any missing residues or side chains were modeled.

The three-dimensional structures of the ligands were retrieved from PubChem. The ligands were energy-minimized and optimized through geometry optimization using molecular mechanics force fields, such as MMFF94. Partial atomic charges were then assigned [31,32]. Reference ligands, including acarbose for α-amylase and an established GLP-1R ligand/agonist, were included as positive controls to evaluate docking reliability.

##### Molecular Docking

To estimate ligand binding modes within experimentally reported binding regions of the target proteins, docking calculations were carried out. Docking was carried out using PyRx 0.8 and AutoDock Vina (version 1.2.6). The coordinates of the co-crystallized ligand were used to create a grid box surrounding the active-site residues. The compounds were imported into PyRx, where energy minimization was performed. Subsequently, they were converted into PDBQT format. Based on binding affinity scores, the top-ranked conformations were chosen and analyzed for ligand–protein interactions, such as hydrophobic contacts, hydrogen bonding, and π–π interactions with pertinent binding-site residues [31,32].

#### 2.8.2. Molecular Dynamics Simulation

Molecular dynamics (MD) simulations were performed using AMBER 24 for 200 ns in order to assess the dynamic stability and conformational behavior of the bound protein–ligand complexes. The selected complexes of α-amylase (PDB ID: 5U3A) and the GLP-1 receptor domain (PDB ID: 6GB1) with lycorine, pratorinine, and palmatine were parameterized using the AMBER ff14SB force field for the proteins and the General AMBER Force Field (GAFF) for the ligands.

In order to neutralize the system and preserve a physiological ionic strength of 0.15 M, Na^+^/Cl^−^ counterions were introduced after each complex was solvated in an explicit TIP3P water box. After removing unfavorable steric contacts using energy minimization, equilibration under NVT and NPT ensembles at 300 K and 1 atm was carried out. Next, a 200 ns production simulation was run for each complex.

To evaluate structural stability, residue-level flexibility, compactness, and persistence of ligand binding, the trajectories were examined using root mean square deviation (RMSD), root mean square fluctuation (RMSF), radius of gyration (RoG/Rgyr), beta-factor, ligand RMSD, and hydrogen-bonding patterns [33,34,35].

#### 2.8.3. Absorption, Distribution, Metabolism, Excretion, and Toxicity Studies

The SwissADME web-based program (https://www.swissadme.ch/index.php; last accessed 11 July 2026) was used to estimate absorption, distribution, metabolism, excretion, and toxicity (ADMET) factors, such as physicochemical qualities, pharmacokinetic properties, lipophilicity, water solubility, and drug-likeness. ProTox-3.0 software (https://comptox.charite.de/protox3/; last accessed 11 July 2026) was used to predict potential Ames toxicity, hepatotoxicity, nephrotoxicity, carcinogenicity, cytotoxicity, and mutagenicity [32].

### 2.9. Statistical Analysis

IBM SPSS Statistics version 19 (IBM Corp., Armonk, NY, USA) and Microsoft Excel 2013 (Microsoft Corporation, Redmond, WA, USA) were used for the statistical analyses. All data are expressed as mean ± standard deviation (SD). Two-way ANOVA followed by Tukey’s HSD post hoc test was used to assess the hypoglycemic effects of different extract doses among treatment groups. Differences were considered statistically significant at *p* < 0.05 [36]. Dose–response curves, obtained by plotting percentage inhibition against concentration, were used to calculate the 50% inhibitory concentration (IC_50_) for both antioxidant and α-amylase inhibitory activities.

## 3. Results

### 3.1. Phytochemical Screening of C. amoenum Extract

Positive results for alkaloids, flavonoids, phenolic compounds, tannins, saponins, and carbohydrates were found in the 80% ethanol bulb extract of *C. amoenum* after phytochemical screening (Table 1). The Salkowski test was also positive, indicating that some constituents are present in glycosidic form.

### 3.2. Thin-Layer Chromatography Profiling of C. amoenum Extracts

**Figure 1:**
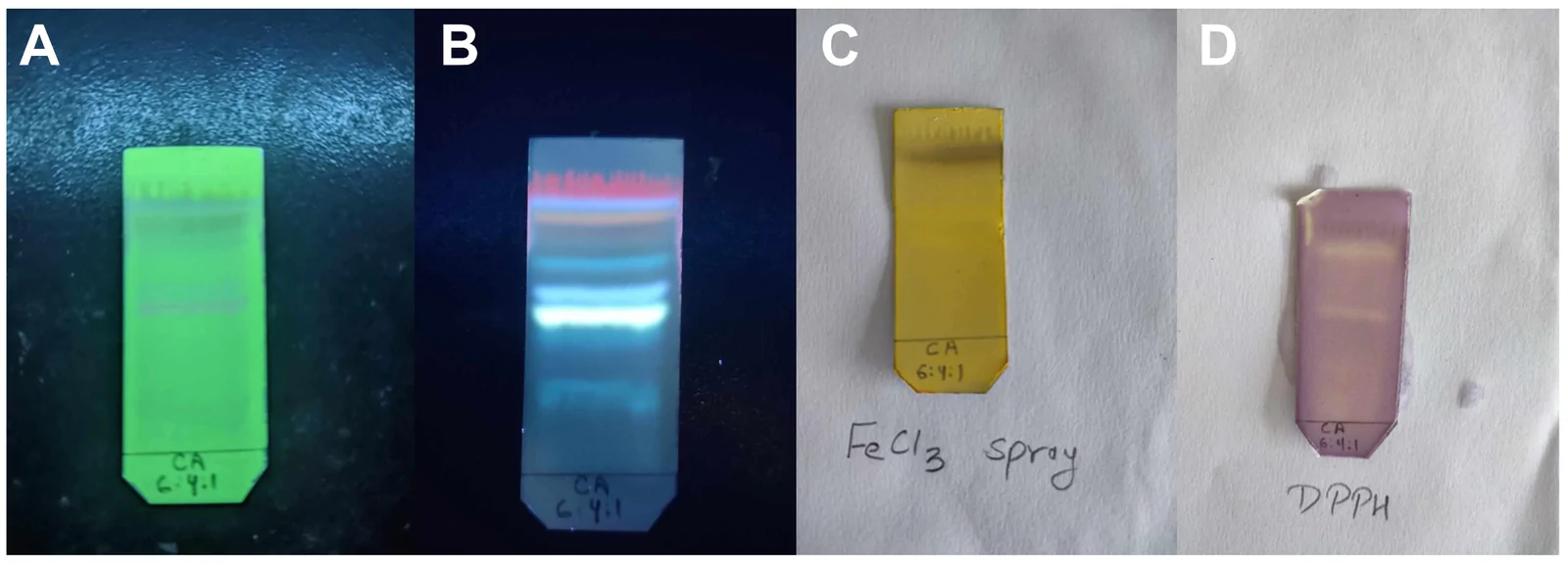
Thin-layer chromatography of the 80% ethanol bulb extract of *C. amoenum* on silica gel 60 F254, developed with chloroform, methanol, and water (7:3:0.5, *v*/*v*/*v*): (**A**) UV light at 254 nm, (**B**) UV light at 365 nm, (**C**) after spraying with 10% FeCl_3_ reagent, indicating the presence of phenolic compounds, and (**D**) after immersion in DPPH solution, in which decolorized zones correspond to radical-scavenging constituents.

The TLC profile of *C. amoenum* bulb extract is shown in Figure 1. The chromatogram observed under UV illumination showed several bands at both 365 and 254 nm. The chromatogram showed yellow, reddish-brown, and black coloration following the application of FeCl_3_ spray and subsequent heating. After immersion of the developed TLC plate in DPPH solution, pale-yellow bands appeared against a violet background, indicating antioxidant-active constituents.

### 3.3. Total Phenolic and Flavonoid Contents of C. amoenum Extract

The extraction yield of the ethanolic extract of the *C. amoenum* bulb was 17.10%. The total phenolic content was 173.38 ± 1.22 mg GAE/g dry extract, while the total flavonoid content was 314.58 ± 0.09 mg quercetin equivalents (QE)/g dry extract. Both the TPC and TFC of the *C. amoenum* bulb extract were estimated using the calibration curve equations (Figures S1 and S2).

### 3.4. LC-MS Profiling

**Figure 2:**
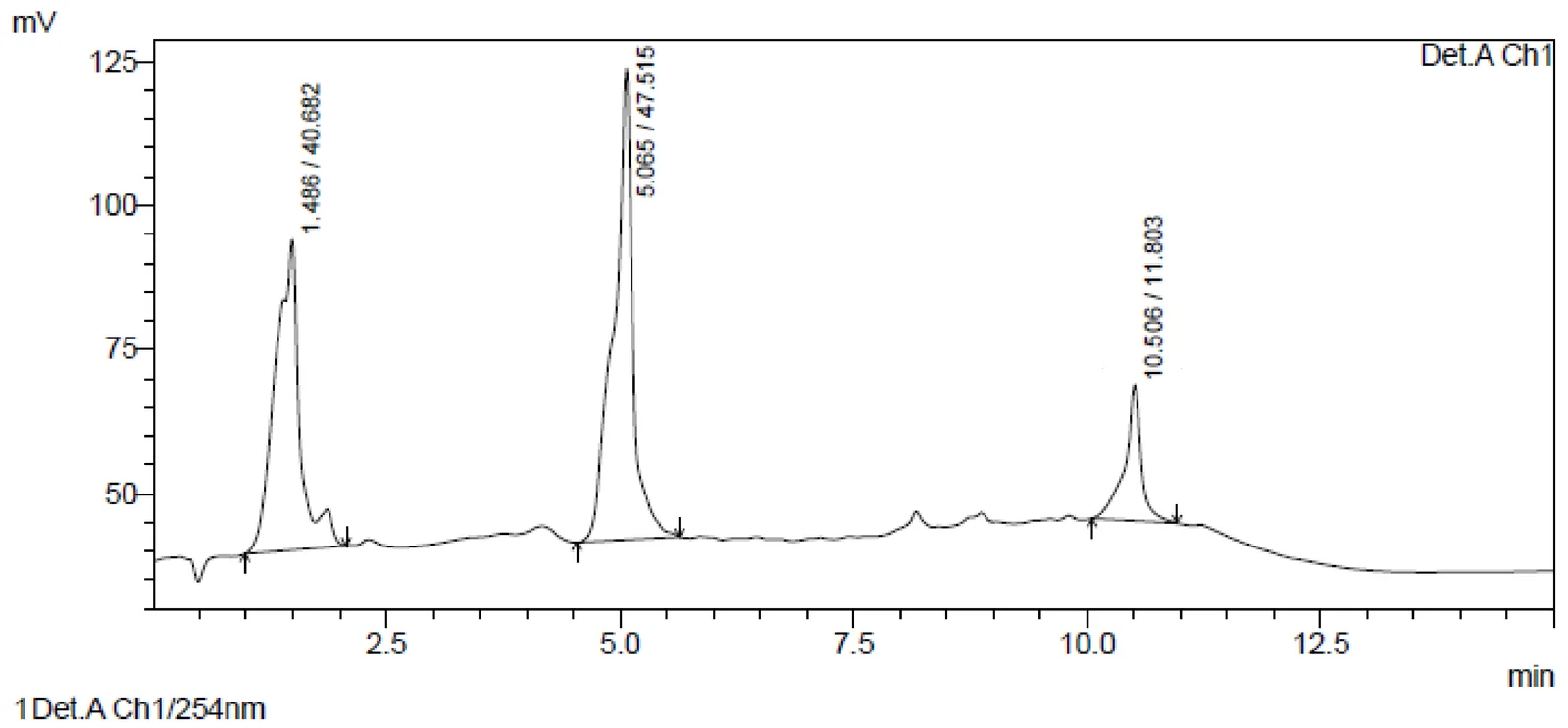
HPLC chromatogram of the 80% ethanol bulb extract of *C. amoenum* at 254 nm.

**Figure 3:**
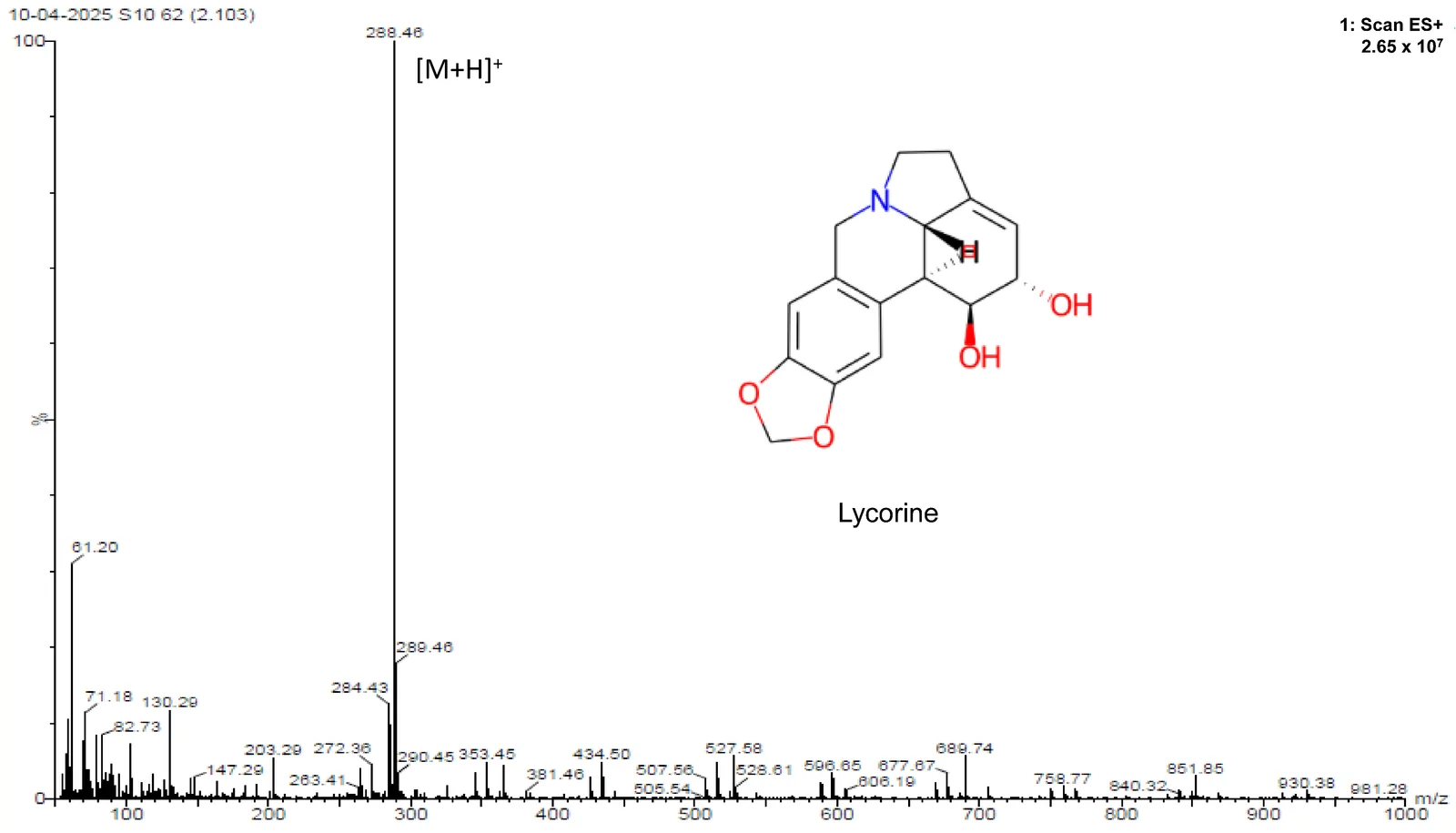
The mass spectrum of Peak 1, with a retention time of 2.1 min, showed a protonated molecular ion at m/z 288.46 [M + H]^+^ in positive-mode ESI. Based on a comparison of the observed m/z value and spectral characteristics with previously reported data for compounds isolated from the same genus, Peak 1 was tentatively annotated as lycorine.

**Figure 4:**
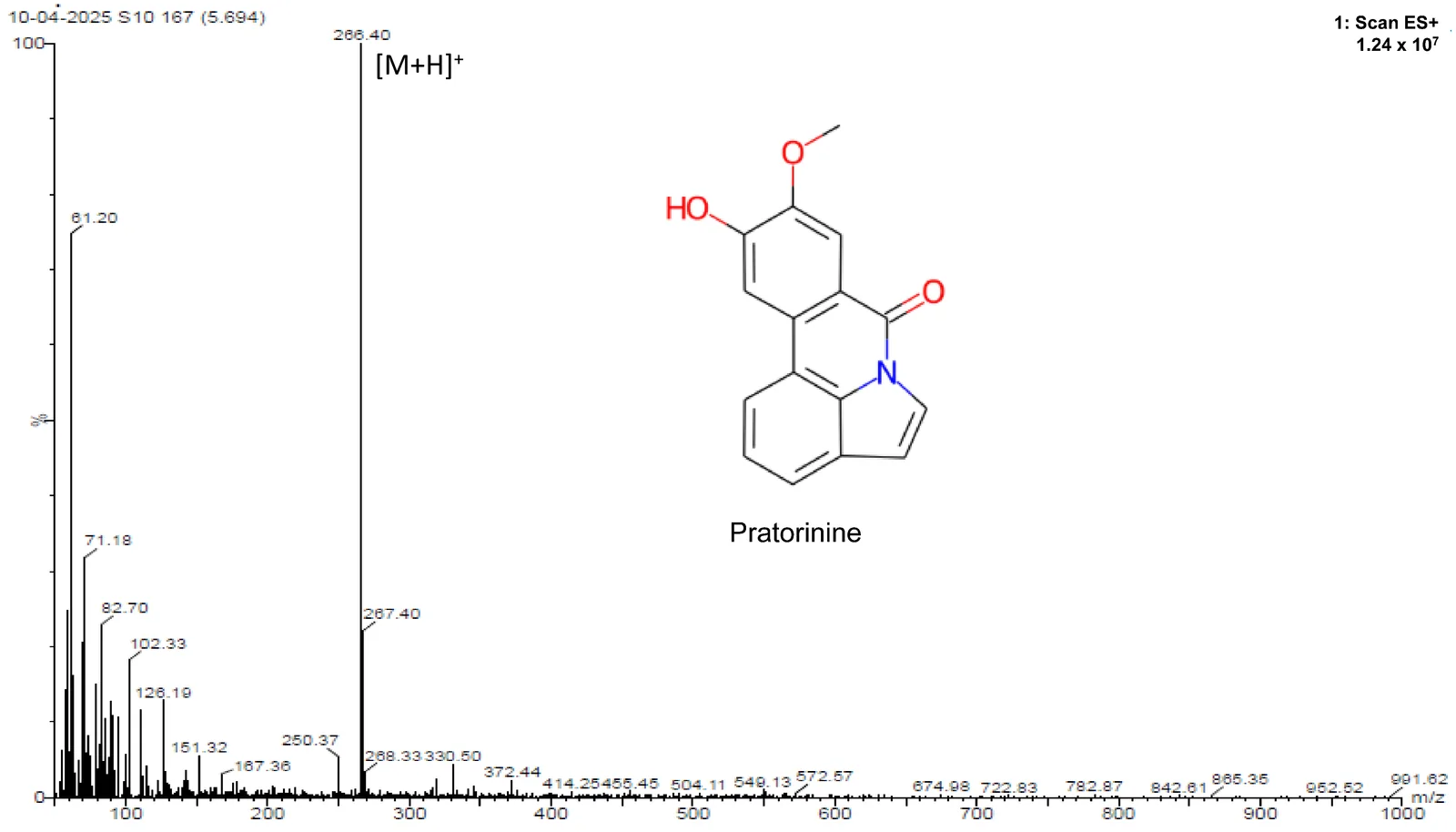
The mass spectrum of Peak 2, with a retention time of 5.6 min, showed a protonated molecular ion at m/z 266.40 [M + H]^+^ in positive-mode ESI. Based on a comparison of the observed m/z value and spectral characteristics with previously reported data for compounds isolated from the same genus, Peak 2 was tentatively annotated as pratorinine.

**Figure 5:**
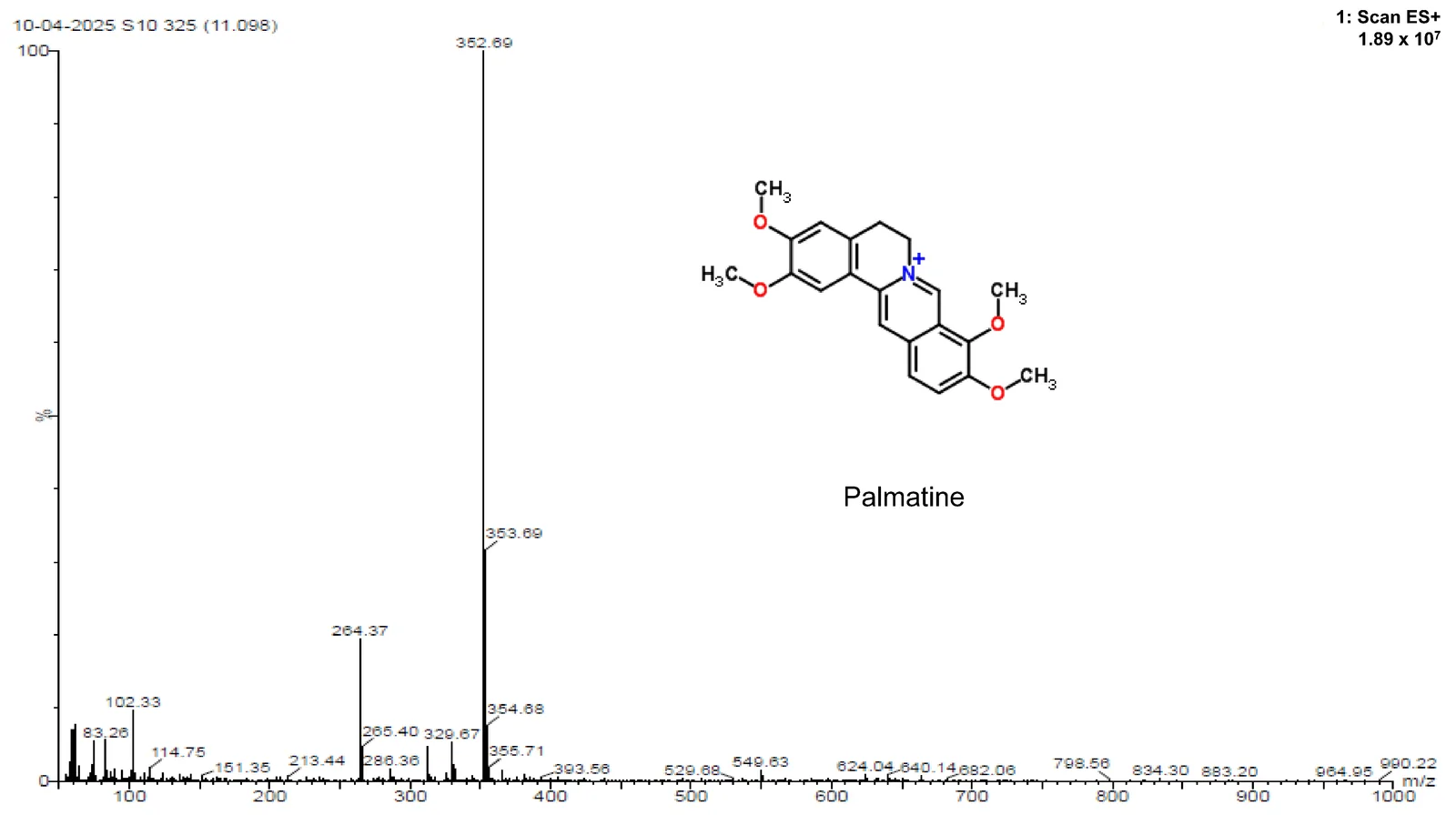
The mass spectrum of Peak 3, with a retention time of 11.09 min, showed a molecular ion at m/z 352.69 [M]^+^ in positive-mode ESI. Based on a comparison of the observed m/z value and spectral characteristics with previously reported data for compounds isolated from the same genus, Peak 3 was tentatively annotated as palmatine.

Three peaks dominated the chromatogram of the 80% ethanol bulb extract of *C. amoenum* (Figure 2). Their retention times, molecular formulae, and observed m/z values are summarized in Table 2, and the corresponding mass spectra are shown in Figure 3, Figure 4 and Figure 5. The peaks were tentatively annotated as lycorine, pratorinine, and palmatine.

### 3.5. In Vitro Antioxidant Activity Using DPPH, NO, H_2_O_2_, and FRAP Method

The DPPH assay, which is based on a stable free radical, is frequently used to evaluate the free radical-scavenging capacity of different compounds as well as the antioxidant capacity of substances. In the DPPH, NO, and H_2_O_2_ tests, the ethanolic extract of *C. amoenum* bulb demonstrated free radical-scavenging activity, with IC_50_ values of 90.98, 865.15, and 951.87 μg/mL, respectively. In the FRAP assay, the ethanolic extract’s reducing power was also noted. These results indicate moderate antioxidant activity of *C. amoenum* bulb extract (Table 3).

### 3.6. α-Amylase Inhibitory Assay

The α-amylase inhibitory activity of *C. amoenum* extract (IC_50_ = 49.31 μg/mL) was slightly stronger than that of the standard drug acarbose (IC_50_ = 51.51 μg/mL), as indicated by its lower IC_50_ value (Table 4).

### 3.7. Oral Acute Toxicity Study

No discernible behavioral changes were observed following oral administration of the ethanolic extract of *C. amoenum* bulb, including variations in locomotion, activity level, hair texture, pupil size, or eating behavior. At a dose of 5000 mg/kg, there were no indications of disease or death.

### 3.8. In Vivo Hypoglycemic Activity

#### 3.8.1. Hypoglycemic Effect in Non-Diabetic Wistar Rats

*C. amoenum* was selected for evaluation of hypoglycemic activity because of its potent α-amylase inhibitory activity. The average blood glucose levels and percentage glycemic changes after the single oral administration of vehicle control, ethanolic extract of *C. amoenum* at doses of 125, 250, and 500 mg/kg, and metformin at 100 mg/kg in non-diabetic Wistar rats over 3 h are shown in Table 5.

Blood glucose levels at different time points were compared with the initial blood glucose level at 0 min, which served as the baseline value for each respective group. The *C. amoenum* 125 mg/kg-treated group showed a gradual reduction in blood glucose levels after extract administration. In contrast, the groups treated with 250 and 500 mg/kg showed marked reductions in blood glucose levels after 30 min, which continued up to 180 min.

#### 3.8.2. Hypoglycemic Effect in Oral Glucose-Induced Hyperglycemic Rats

Table 6 displays the oral glucose tolerance test results. Blood glucose levels were monitored at 0, 30, 60, 120, and 180 min in glucose-loaded rats to assess the hypoglycemic effect of the ethanolic extract of *C. amoenum* bulb. Blood glucose levels increased at 30 min after glucose administration (2 g/kg) in all groups and then gradually decreased.

Changes in blood glucose levels were compared with baseline values. Fasting blood glucose levels at 0 min ranged from 112 to 165 mg/dL across all groups. The metformin (100 mg/kg) and *C. amoenum* (500 mg/kg)-treated groups showed reductions in blood glucose levels from 60 to 180 min. The *C. amoenum* 500 mg/kg group showed reductions at 60, 120, and 180 min compared with baseline values. Therefore, the 500 mg/kg dose produced a greater numerical reduction in blood glucose levels; however, direct superiority over metformin should be interpreted cautiously unless supported by appropriate statistical comparisons between treatment groups.

### 3.9. In Silico Studies

#### 3.9.1. Molecular Docking

The binding mechanisms and affinities of the three *C. amoenum* alkaloids identified by LC-MS against certain antidiabetic target proteins, such as α-amylase and the GLP-1 receptor (GLP-1R), were predicted using molecular docking. The docking results should be interpreted as computational predictions and require further experimental validation.

The co-crystallized ligand and binding-site residues were mapped into the three-dimensional crystal structures of the target proteins using BIOVIA Discovery Studio for active-site analysis [37]. This visualization identified key catalytic residues of α-amylase, including ASP300, GLU233, and ASP197, as well as GLY132 and other interacting residues in GLP-1R identified from the docking analysis. Within the enzyme or receptor binding site, these residues play a role in molecular interactions and ligand recognition [13]. The three-dimensional space for site-specific molecular docking investigations was defined in part by these discoveries.

**Figure 6:**
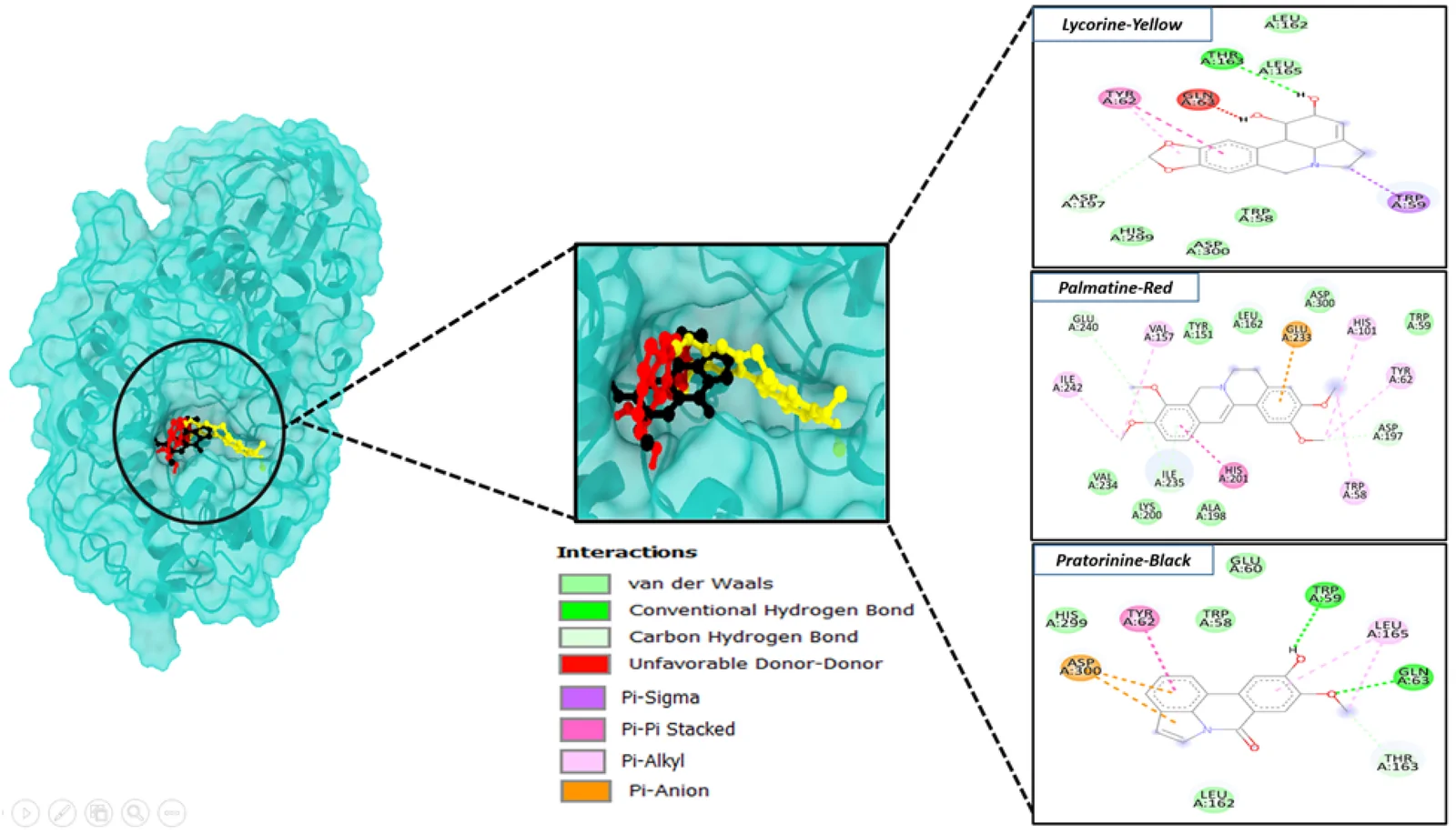
Two-dimensional and three-dimensional interactions of *C. amoenum* constituents within the active site of human pancreatic α-amylase.

**Figure 7:**
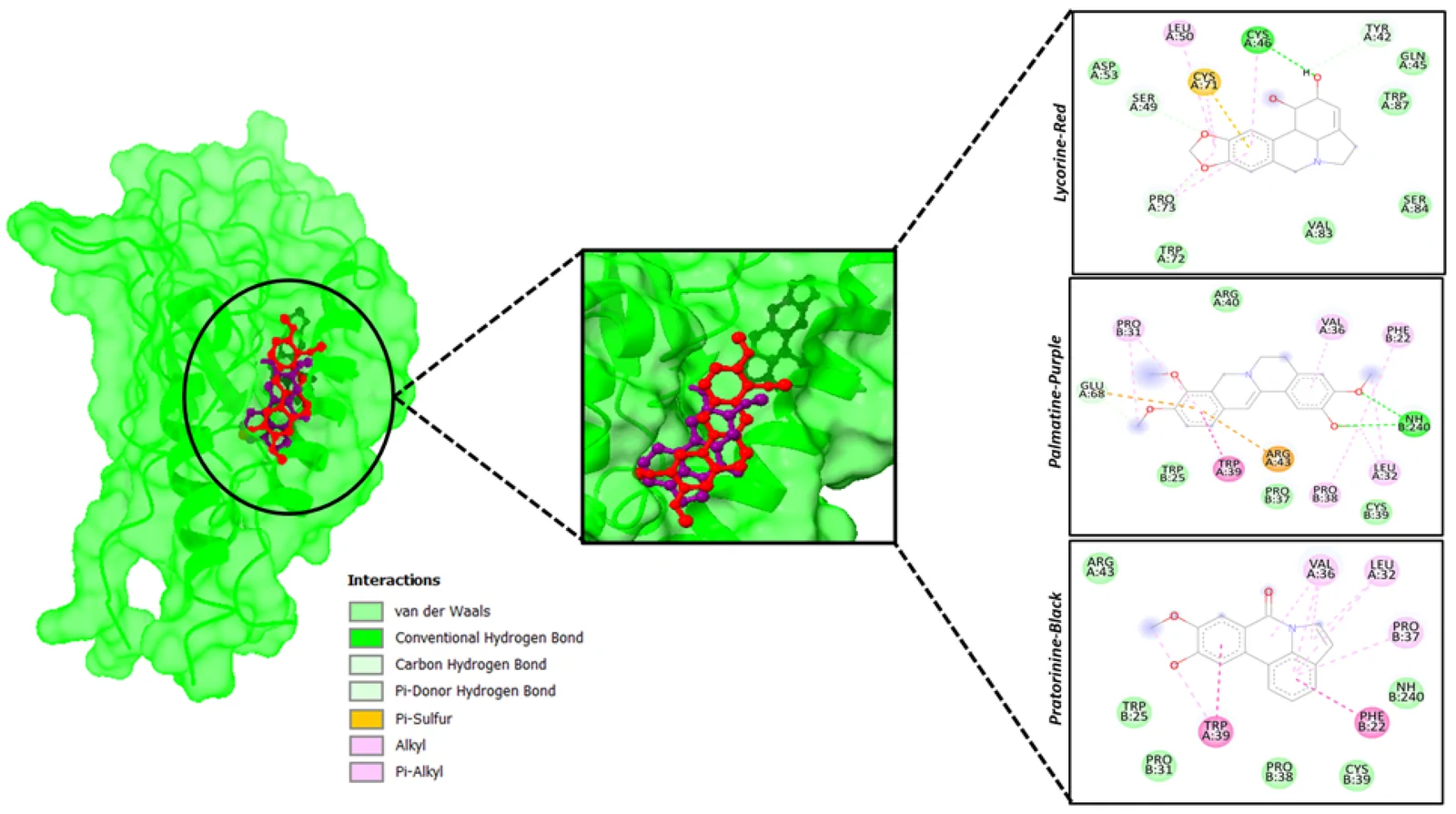
Two-dimensional and three-dimensional interactions of *C. amoenum* constituents within the active site of the GLP-1 receptor.

Docking results prioritized ligands with strong negative binding affinity scores, favorable hydrogen bonding, and short interaction distances. Among the tested constituents, pratorinine showed the strongest predicted binding affinity, with docking scores of −8.2 kcal/mol against α-amylase and −7.0 kcal/mol against GLP-1R, as summarized in Table 7. Conventional hydrogen bonds, carbon–hydrogen bonds, π–σ interactions, π–π T-shaped interactions, π–donor hydrogen bonds, π–alkyl interactions, and π–π stacking interactions were among the various ligand–protein interactions identified by molecular interaction analysis, with hydrogen bonding being the most common. These interactions suggest the possible formation of stable ligand–enzyme or ligand–receptor complexes [38] (Figure 6 and Figure 7).

#### 3.9.2. Molecular Dynamics Simulations

Using the AMBER 24 package, 200 ns molecular dynamics (MD) simulations were run to assess the dynamic stability and conformational behavior of the docked complexes. The overall stability, flexibility, and conformational adaptation of the *C. amoenum* constituents–α-amylase and *C. amoenum* constituents–GLP-1R complexes were evaluated during the simulation period using RMSD and RMSF analyses.

To determine whether the docked complexes of the three main components of *C. amoenum*—lycorine, pratorinine, and palmatine—remained stable under simulated physiological conditions, molecular dynamics simulations were carried out. The docking results showed that all three ligands interacted with catalytic or active-site residues of α-amylase and GLP-1R, with pratorinine showing the strongest docking affinity against α-amylase (−8.2 kcal/mol; ASP300) and GLP-1R (−7.0 kcal/mol; GLY132). Lycorine showed binding scores of −8.0 kcal/mol against α-amylase and −6.6 kcal/mol against GLP-1R, while palmatine showed binding scores of −7.7 kcal/mol and −6.4 kcal/mol, respectively. In order to investigate the dynamic stability of these complexes beyond the static docking positions, 200 ns MD simulations were performed for all of them.

For the α-amylase complexes, the RMSD profiles indicated stable protein–ligand behavior throughout the simulation period. The pratorinine–α-amylase and palmatine–α-amylase complexes showed lower average RMSD values of approximately 1.77 Å and 1.80 Å, respectively, while the lycorine–α-amylase complex showed a slightly higher average RMSD value of approximately 2.18 Å. Although transient deviations were observed, the RMSD values did not indicate major conformational drift, suggesting that all three ligands remained well accommodated within the α-amylase binding cavity during the 200 ns simulation. Among these complexes, pratorinine showed the most favorable RMSD profile, supporting its strong docking score and stable interaction with the catalytic residue ASP300.

The RMSF profiles further supported the stability of the α-amylase complexes. Most residues showed low-to-moderate fluctuation, whereas higher peaks were mainly observed in flexible loop regions or surface-exposed regions. Importantly, the catalytic residues ASP197, GLU233, and ASP300 showed comparatively lower fluctuations than the surface-exposed regions, suggesting that ligand binding stabilized the catalytic pocket. The radius of gyration (RoG/Rgyr) values also remained within a narrow range for all three α-amylase complexes, with average values of approximately 23.30–23.55 Å, indicating that the overall compactness and folded state of α-amylase were maintained during the simulation. The beta-factor profile showed localized flexibility in selected loop regions, but this did not affect the global stability of the complexes.

For the GLP-1R complexes, the RMSD and RMSF profiles showed comparatively greater flexibility than those of the α-amylase systems. The average RMSD values were approximately 2.88 Å for lycorine, 3.24 Å for palmatine, and 3.35 Å for pratorinine. Pratorinine showed a transient rise in RMSD during the later phase of the simulation, suggesting conformational rearrangement within the GLP-1R binding region rather than complete instability. The RMSF plots showed higher residue-level mobility, particularly in flexible terminal or loop regions, whereas active-site residues such as LEU123 showed comparatively lower fluctuation. The RoG profiles of the GLP-1R complexes remained stable, with average values of approximately 22.30–22.44 Å, indicating that the receptor domain maintained its compactness despite local flexibility. Thus, the GLP-1R complexes appeared dynamically acceptable but more flexible than the α-amylase complexes.

**Figure 8:**
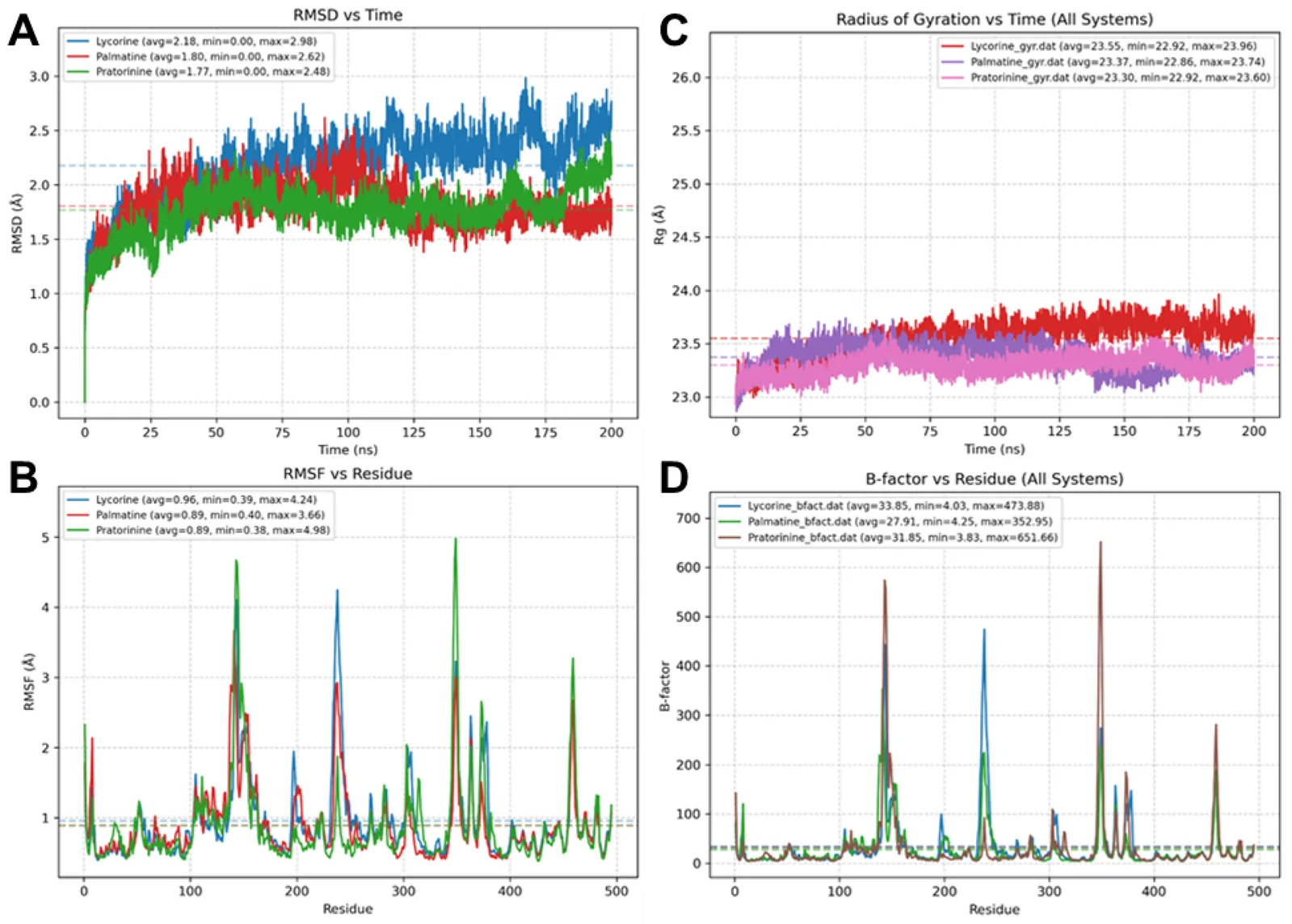
(**A**) Root mean square deviation (RMSD), (**B**) root mean square fluctuation (RMSF), (**C**) radius of gyration, and (**D**) beta-factor analysis of the *C. amoenum* constituents–α-amylase complexes.

**Figure 9:**
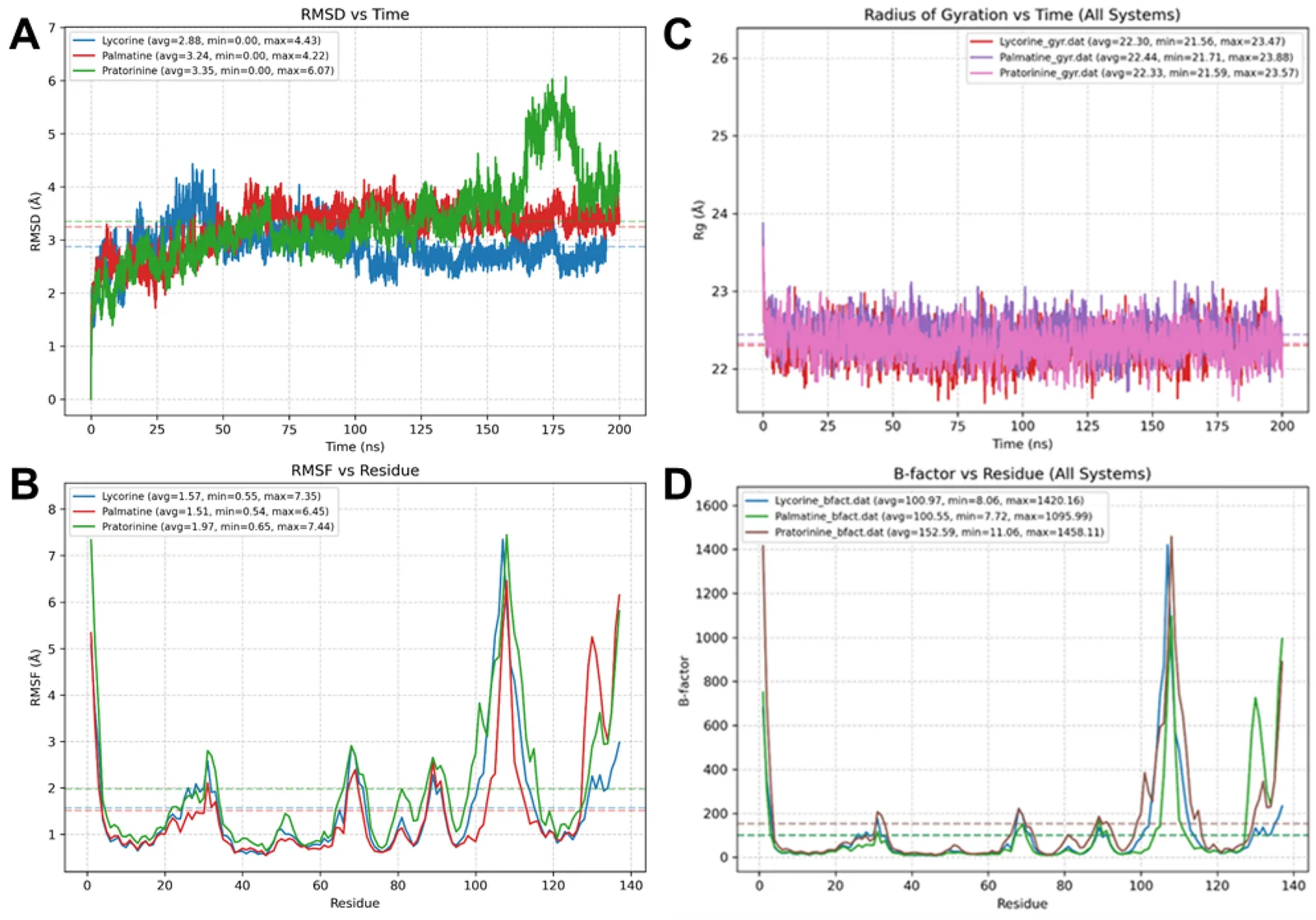
(**A**) Root mean square deviation (RMSD), (**B**) root mean square fluctuation (RMSF), (**C**) radius of gyration, and (**D**) beta-factor analysis of the compound–human pancreatic α-amylase complexes.

**Figure 10:**
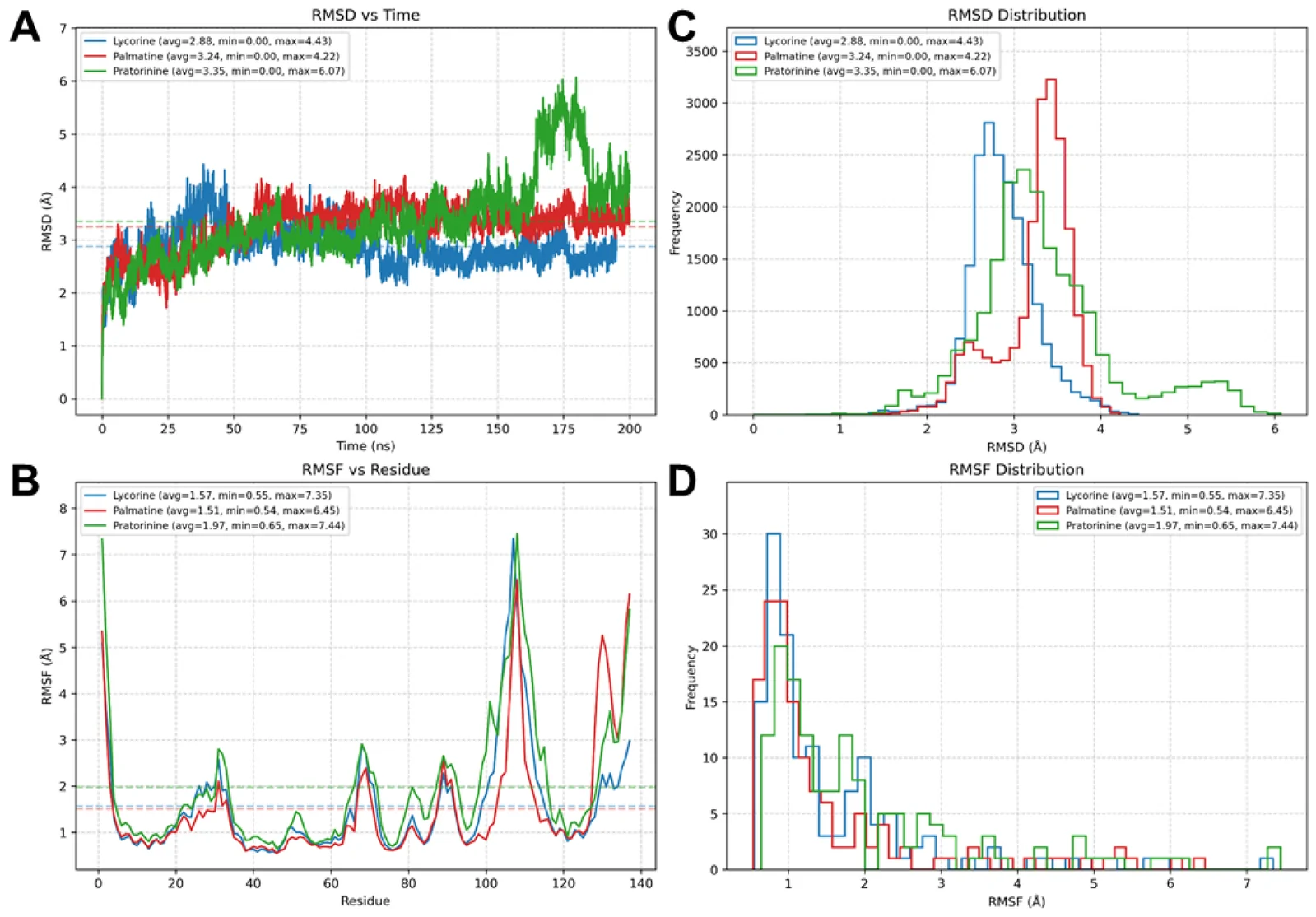
(**A**) Root mean square deviation (RMSD), (**B**) root mean square fluctuation (RMSF), (**C**) RMSD distribution, and (**D**) RMSF distribution analysis of the compound–human GLP-1 receptor complexes.

**Figure 11:**
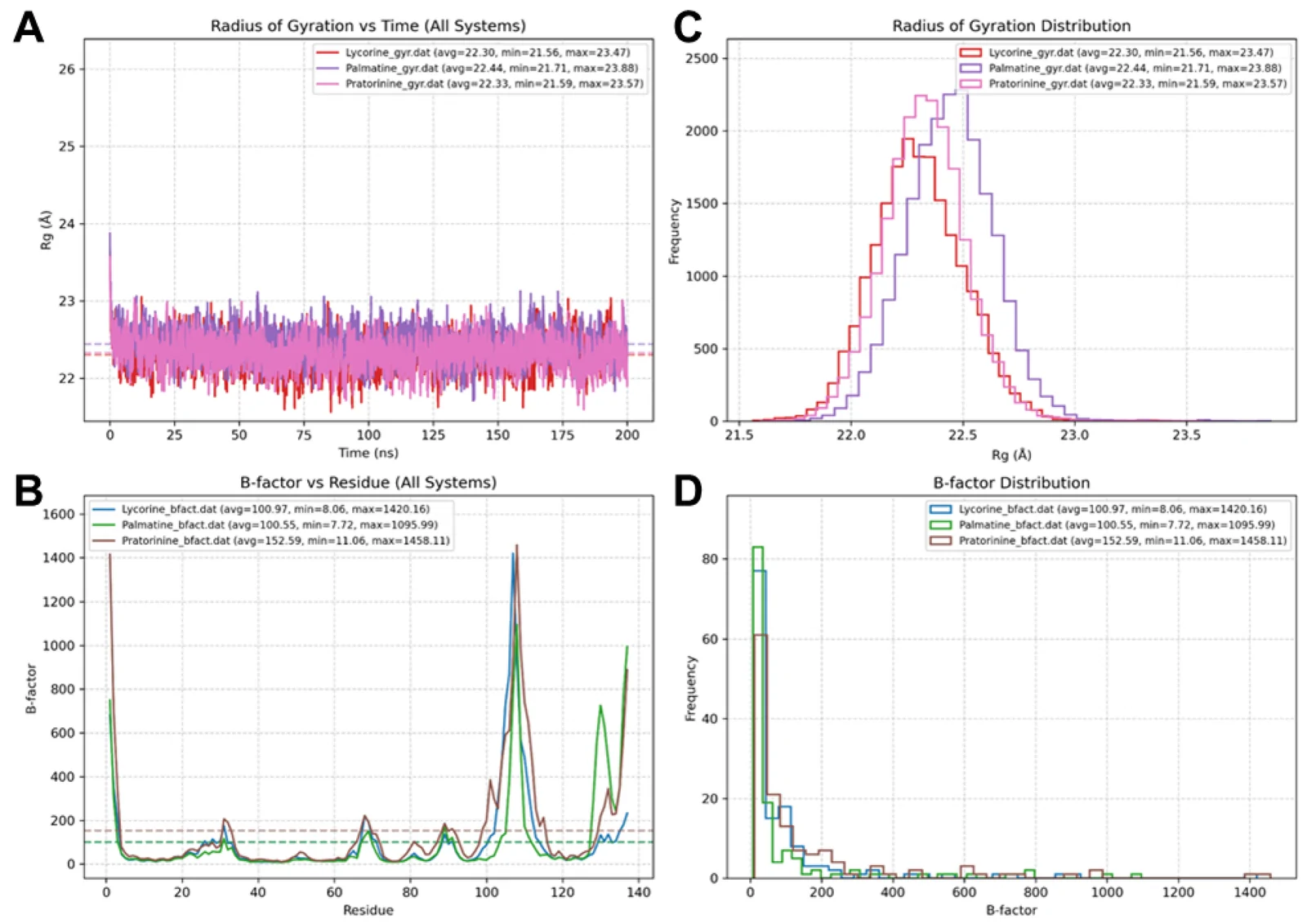
(**A**) Radius of gyration, (**B**) beta factor, (**C**) radius of gyration distribution, and (**D**) beta-factor distribution of the compound–human GLP-1 receptor complexes.

Overall, the MD simulation results support the docking findings by showing that lycorine, pratorinine, and palmatine can form stable complexes with α-amylase and moderately stable complexes with GLP-1R. The α-amylase complexes were more stable, compact, and less fluctuating than the GLP-1R complexes, suggesting that α-amylase inhibition may be the major molecular mechanism contributing to the antidiabetic effect of *C. amoenum*. The comparatively stronger stability of pratorinine and palmatine with α-amylase agrees with the in vitro α-amylase inhibitory activity reported for the extract, while GLP-1R interaction suggests a possible secondary mechanism that requires further experimental validation. The MD analyses also showed that the α-amylase catalytic residues had lower RMSF variability, RoG values remained stable, and GLP-1R complexes displayed acceptable stabilization patterns with stable ligand positioning (Figure 8, Figure 9, Figure 10 and Figure 11).

#### 3.9.3. In Silico ADMET Studies

The SwissADME online tool and ProTox-3.0 were used to assess the absorption, distribution, metabolism, excretion, and toxicity (ADMET) profiles of the three compounds that were tentatively annotated in this investigation. The results are shown in Table 8.

The in silico ADMET profiling of the major constituents of *C. amoenum* indicated generally acceptable pharmacokinetic and drug-likeness properties across the evaluated compounds. Among the identified molecules, pratorinine demonstrated the most favorable overall safety profile, with comparatively favorable predicted pharmacokinetic characteristics and a comparatively lower toxicity risk, including an estimated LD_50_ of 1000 mg/kg, corresponding to toxicity Class 4. In contrast, lycorine and palmatine showed moderate pharmacokinetic and toxicity liabilities. Overall, the ADMET findings suggest that pratorinine may serve as the most promising lead compound because of its balanced efficacy-related binding potential and comparatively safer predicted toxicological profile.

## 4. Discussion

The present study evaluated α-amylase inhibition, in vivo glucose-lowering activity, molecular docking, molecular dynamics simulation, and ADMET properties of the 80% ethanolic bulb extract of *C. amoenum*, demonstrating its hypoglycemic potential. Oxidative stress and inflammation contribute to chronic diseases, including cardiovascular disease, diabetes, cancer, and neurodegeneration, through damage to lipids, proteins, and DNA, as well as through endothelial dysfunction, insulin resistance, and altered cell signaling [39,40]. Antioxidants may therefore delay disease progression by reducing oxidative stress and strengthening endogenous defense systems [41]. Medicinal plants are important sources of phenolics and flavonoids, which are recognized for their antioxidant and disease-preventive properties [42].

Phytochemical screening of *C. amoenum* species revealed phenols, flavonoids, saponins, alkaloids, glycosides, and carbohydrates, consistent with previous reports [9,43]. TLC profiling supported these findings, showing multiple separated compounds comparable with earlier studies (Figure 1) [44]. Black spots on the TLC plate indicated saccharides, whereas brownish-gray and yellow spots suggested phenols and flavonoids [45]. White or yellow decolorized zones after DPPH treatment indicated antioxidant-active constituents, as reported by Wang et al. [46]. Quantitative analysis showed an extraction yield of 17.10%, with total phenolic and flavonoid contents of 173.38 ± 1.22 mg GAE/g and 314.58 ± 0.09 mg QE/g dry extract weight, respectively, comparable to the findings of Baral et al. [47]. These results point to a significant phenolic content that could support biological activities such as antioxidant, anti-inflammatory, and anticancer effects [48,49,50].

DPPH, nitric oxide, hydrogen peroxide, and FRAP assays were used to evaluate the antioxidant activity of the ethanolic extract. The extract showed DPPH radical-scavenging activity, with an IC_50_ of 90.98 µg/mL, comparable to that reported by Baral et al. [47] and stronger than the IC_50_ of 661.76 µg/mL reported by Tiwari et al. [8]. To our knowledge, NO, H_2_O_2_, and FRAP-based antioxidant activities have not previously been reported for *C. amoenum*. The extract showed moderate NO-scavenging activity, with an IC_50_ of 865.15 µg/mL compared with curcumin at 162.79 µg/mL, and H_2_O_2_-scavenging activity, with an IC_50_ of 951.87 µg/mL compared with ascorbic acid at 353.96 µg/mL. In the FRAP assay, the extract showed reducing power with an absorbance of 0.233 ± 0.0020 at 700 nm, whereas ascorbic acid showed an absorbance of 1.07 ± 0.006. These findings imply that *C. amoenum* may act as a free electron donor, stabilize free radicals, and interrupt radical chain reactions [51].

Under experimental test conditions, the extract also demonstrated considerable α-amylase inhibitory activity, with an IC_50_ of 49.31 µg/mL as opposed to 51.51 µg/mL for acarbose. This effect may be linked to its phenolic content and agrees with Ghane et al., who reported α-amylase inhibition by a methanolic extract of *Crinum* species, with an IC_50_ of 60.30 µg/mL [10]. Phenolic compounds may reduce blood glucose by inhibiting carbohydrate digestion and intestinal glucose absorption, stimulating insulin secretion, regulating hepatic glucose metabolism, and enhancing glucose uptake in insulin-sensitive tissues [52]. Enzyme inhibition may also be facilitated by other phytochemicals found in the extract, such as alkaloids, flavonoids, glycosides, saponins, and phenolic compounds. The acute toxicity testing showed no mortality up to 5000 mg/kg, indicating that the oral LD_50_ of the ethanolic extract is higher than 5000 mg/kg. This places the extract in Category 5 under the Globally Harmonized System of Classification and Labeling of Chemicals, indicating the least hazardous category following oral ingestion.

The in vivo hypoglycemic effect of *C. amoenum* bulb extract was evaluated in normoglycemic and glucose-induced hyperglycemic rats. In non-diabetic Wistar rats, blood glucose decreased modestly 30 min after oral administration. At 500 mg/kg, the extract produced the greatest numerical reduction in blood glucose at 120 and 180 min. At 120 min, metformin 100 mg/kg and *C. amoenum* 500 mg/kg reduced blood glucose by 23.10% and 35.78%, respectively, indicating stronger numerical activity at the highest extract dose. However, direct superiority over metformin should be interpreted cautiously unless supported by appropriate statistical comparisons between treatment groups. This effect may be associated with phenolic compounds and glycosides, as supported by TLC and quantitative analyses. Baral et al. reported cardiac glycosides in the ethanolic extract [47], while Capasso et al. demonstrated their beneficial effects in diabetes [53]. Polyphenols, flavonoids, alkaloids, and glycosides are commonly reported in antidiabetic plants [54], and similar constituents were present in *C. amoenum*. Since oral hypoglycemic agents generally reach peak plasma concentrations within 1–3 h [55], the OGTT result is pharmacologically relevant. In the OGTT, *C. amoenum* extract at 500 mg/kg reduced blood glucose by 19.21% at 60 min, compared with 18.48% for metformin at the same time point. Thus, peak activity was observed at 60 min for both treatments, consistent with previous findings [56]. Flavonoids, saponins, steroids, terpenoids, and other constituents may act individually or synergistically to produce this hypoglycemic response.

In silico studies supported the experimental findings. LC-MS tentatively annotated three compounds—pratorinine, lycorine, and palmatine—based on spectral data and previous reports from related species. Docking against human α-amylase (PDB ID: 5U3A) and GLP-1R (PDB ID: 6GB1) showed distinct binding affinities. Pratorinine displayed the lowest binding energies of −8.2 and −7.0 kcal/mol against α-amylase and GLP-1R, respectively, indicating favorable ligand–target complex formation [57]. It interacted with ASP300 in 5U3A and GLY132 in 6GB1, suggesting potential interaction with both targets. However, GLP-1R activity requires experimental validation and cannot be concluded from docking alone. The 2D and 3D interaction diagrams (Figure 6 and Figure 7) showed stable complexes dominated by hydrogen bonding, along with π–π stacking, π–alkyl, and carbon–hydrogen interactions. GLP-1R interaction suggests a possible molecular association that requires experimental validation to establish receptor activation or insulinotropic effects. Lycorine showed binding energies of −8.0 kcal/mol against α-amylase and −6.6 kcal/mol against GLP-1R, interacting with ASP197 and GLU26, respectively. As an Amaryllidaceae alkaloid [11,14], lycorine has reported antimalarial [14], cytotoxic [15], anti-acetylcholinesterase [10], and antiviral activities [14]. Palmatine showed binding energies of −7.7 and −6.4 kcal/mol against α-amylase and GLP-1R, respectively. Overall, stronger binding to 5U3A than to 6GB1 suggests that α-amylase inhibition is the primary antidiabetic mechanism, consistent with the extract’s α-amylase IC_50_ of 49.31 µg/mL compared with acarbose at 51.51 µg/mL.

The stability of these *C. amoenum* constituent–protein complexes was further evaluated through 200 ns molecular dynamics simulations using AMBER 24. RMSD analysis showed that the pratorinine–α-amylase and palmatine–α-amylase complexes remained stable, with fluctuations below 2.0 Å, indicating stable ligand retention without major conformational drift. RMSF analysis showed lower variability in catalytic residues ASP300, GLU233, and ASP197 than in surface-exposed loops, suggesting stabilization of the α-amylase catalytic pocket. Radius of gyration values remained within a narrow range, confirming preserved enzyme compactness. For GLP-1R complexes, RMSD and RMSF profiles showed greater flexibility, with average RMSD values of approximately 2.88 Å for lycorine, 3.24 Å for palmatine, and 3.35 Å for pratorinine, while radius of gyration values remained stable at approximately 22.30–22.44 Å. These findings indicate that GLP-1R complexes were dynamically acceptable but more flexible than α-amylase complexes. Therefore, GLP-1R involvement should be interpreted as a possible supportive mechanism rather than confirmed agonistic activity.

The pharmacokinetic and safety properties of the identified compounds were further clarified using ADMET profiling. With molecular weights below 500 Da, hydrogen bond acceptors ≤10, hydrogen bond donors ≤5, and appropriate logP values, all compounds complied with Lipinski’s Rule of Five. Pratorinine showed a favorable logP of 2.288, consistent with hydrophobic binding interactions. Lycorine had a TPSA of 62.16 Å^2^ and a logP of −0.268, suggesting higher hydrophilicity that may limit oral bioavailability despite extensive hydrogen bonding. Palmatine showed intermediate properties, with a logP of 2.893 and predicted human intestinal absorption above 30%. However, all compounds showed low Caco-2 permeability values, ranging from −5.148 to −4.822, suggesting that formulation strategies may be needed to improve oral absorption. Clearance values of 6.257–8.128 mL/min/kg, half-lives of 1.078–2.987 h, and volumes of distribution of 0.485–1.314 L/kg indicate relatively rapid elimination and moderate tissue distribution. Toxicity prediction classified lycorine and palmatine as Class 3 toxic compounds, with LD_50_ values of 230 and 200 mg/kg, respectively, whereas pratorinine showed the widest safety margin, with an LD_50_ of 1000 mg/kg and Class 4, slightly toxic, status.

Overall, the computational, in vitro, and in vivo findings provide mechanistic support for the potential antidiabetic activity of *C. amoenum*. The combined actions of lycorine, pratorinine, and palmatine may explain the strong α-amylase inhibition and observed hypoglycemic effects, which are also consistent with the docking and molecular dynamics simulation results. However, the relative abundance of these alkaloids in the extract was not determined, and other constituents detected by qualitative screening may contribute to the observed activity. The contribution of any single compound therefore remains to be established by fractionation and testing of purified material. In the OGTT, the 500 mg/kg dose produced maximal activity at 60 min, while in normoglycemic rats, the maximum reduction was observed at 120 min. These findings are consistent with the predicted short half-lives of 1–3 h and suggest potential usefulness in postprandial glucose regulation. The 200 ns MD simulations confirmed that the three compounds form stable and compact complexes, particularly with α-amylase. Compared with GLP-1R complexes, α-amylase systems showed lower RMSD, reduced RMSF fluctuations around catalytic residues, and a stable radius-of-gyration profiles, supporting α-amylase inhibition as the most plausible primary mechanism. Taken together, these findings indicate that *C. amoenum* is a promising source of multimodal antidiabetic lead molecules.

The three alkaloids were annotated tentatively from retention time and nominal m/z data obtained on a single-quadrupole instrument, without authentic standards or NMR confirmation, and their concentrations in the extract were not determined. The biological evaluation used a crude extract and a single oral dose, and the in vivo work was limited to normoglycemic and glucose-loaded rats followed for 180 min, without a chemically induced diabetic model, repeated dosing, insulin or HbA1c measurement, or histological examination. Finally, the docking, molecular dynamics, and ADMET results are computational predictions. The GLP-1R findings in particular indicate a possible molecular association and cannot be interpreted as receptor activation or as evidence of an insulinotropic effect without cell-based confirmation. The isolation and identification of bioactive compounds from plant-based medicines, the clarification of their molecular mechanisms of action, and the confirmation of their safety and therapeutic efficacy through well-planned in vivo and clinical trials should be the main goals of future research. In parallel, developing balanced intellectual property frameworks will be essential to promote innovation while ensuring equitable access, affordability, and sustainable utilization of plant-derived therapeutic resources in developing countries [58]. To confirm the effectiveness and safety of *C. amoenum* for diabetes management, further histological studies, bioassay-guided fractionation, isolation of active components, formulation optimization, and toxicity assessments are needed.

## 5. Conclusions

Alkaloids, flavonoids, phenolic compounds, tannins, saponins, and carbohydrates were detected in the 80% ethanolic bulb extract of *C. amoenum*. The TPC and TFC were 173.38 ± 1.22 mg GAE/g and 314.58 ± 0.09 mg QE/g of dry extract, respectively. The extract demonstrated reducing power in the FRAP assay, scavenged DPPH, nitric oxide, and hydrogen peroxide radicals, and inhibited porcine pancreatic α-amylase with an IC_50_ of 49.31 µg/mL under the assay conditions. The 500 mg/kg dose reduced blood glucose by 35.78% at 120 min in normoglycemic rats and by 19.21% at 60 min in glucose-loaded rats, and no mortality was observed at 5000 mg/kg. LC-MS tentatively annotated lycorine, pratorinine, and palmatine, and among these, pratorinine showed the strongest docking affinity, the most stable α-amylase complex over 200 ns, and the widest predicted safety margin. Since the individual alkaloids were neither quantified nor isolated, the activity of the extract cannot be assigned to a single constituent, and pratorinine is reported here as an in silico lead only. Bioassay-guided fractionation followed by structural confirmation and testing of purified compounds is the necessary next step.

## Figures and Tables

**Table 1:** Phytochemical screening of *C. amoenum* bulb extract.

Secondary Metabolite	Test Name	Inference
Alkaloids	Wagner test	+
Flavonoids	Alkaline reagent test	+
Phenols	Sodium hydroxide test	+
Tannins	Ferric chloride test	+
Glycosides	Salkowski’s test	+
Saponins	Lead acetate test	+
Carbohydrates	Fehling test	+

Note: +, presence.

**Table 2:** LC-MS-based tentative annotation of constituents in the 80% ethanol bulb extract of *C. amoenum*.

Peak No.	RT (min)	Identification	Molecular Formula	Molecular Weight (MW)	Experimental m/z (ESI+)
1	2.1	Lycorine	C_16_H_17_NO_4_	287.31	288.46 [M + H]^+^
2	5.6	Pratorinine	C_16_H_11_NO_3_	265.27	266.40 [M + H]^+^
3	11.1	Palmatine	C_21_H_22_NO_4_^+^	352.40	352.69 [M]^+^

**Table 3:** Antioxidant activity of the extract using DPPH, NO, H_2_O_2_, and FRAP methods.

Antioxidant Assay	Sample	Antioxidant and Scavenging Activity at Different Concentrations (µg/mL)					IC_50_ (µg/mL)
DPPH scavenging (%)	*C. amoenum* Ascorbic acid	2.5	5	7.5	15	30	
		1.2 ± 0.17	5.37 ± 0.31	9.56 ± 0.38	12.7 ± 0.75	19.53 ± 0.17	90.98
		22.2 ± 0.5	48.4 ± 2.1	54.5 ± 1.0	61.34 ± 0.3	76.28 ± 2.0	3.96
NO scavenging (%)	*C. amoenum* Curcumin	10	100	250	500	1000	
		3.38 ± 0.08	7.61 ± 0.87	16.33 ± 1.16	27.03 ± 0.76	58.73 ± 0.47	865.15
		33.89 ± 0.7	9.28 ± 0.6	22.11 ± 0.6	37.33 ± 2.9	91.09 ± 1.34	162.79
H_2_O_2_ scavenging (%)	*C. amoenum* Ascorbic acid	-	100	250	500	1000	
		-	8.58 ± 0.49	17.57 ± 0.34	30.1 ± 0.45	51.6 ± 6.15	951.87
		-	28.7 ± 0.2	42.12 ± 0.3	61.89 ± 4.4	94.3 ± 1.7	353.96
FRAP (Abs_700nm_)	*C. amoenum* Ascorbic acid	12.5	25	50	75	100	
		0.234 ± 0.007	0.388 ± 0.008	0.545 ± 0.005	0.797 ± 0.006	1.07 ± 0.006	

All data are expressed as mean ± standard deviation (SD) (n = 3). Abbreviations: DPPH: 2,2-diphenyl-1-picrylhydrazyl. NO: Nitric oxide. H_2_O_2_: Hydrogen peroxide. FRAP: Ferric ion reducing antioxidant power. Abs: UV absorbance. IC_50_: Half-maximal inhibitory concentration.

**Table 4:** α-amylase inhibitory activity of 80% ethanol bulb extract of *C. amoenum*.

Sample	α-Amylase Inhibitory Activity (%) at Different Concentrations					
	50 µg/mL	100 µg/mL	250 µg/mL	500 µg/mL	1000 µg/mL	IC_50_ Value (µg/mL)
*C. amoenum*	47.03 ± 1.33	52.99 ± 1.51	58.25 ± 0.24	68.5 ± 1.49	85.93 ± 2.28	49.31
Acarbose	16.06 ± 0.49	14.31 ± 0.36	21.88 ± 0.5	35.04 ± 0.24	53.75 ± 1.39	51.51

All data are expressed as mean ± standard deviation (SD), n = 3.

**Table 5:** Effect of *C. amoenum* bulb extract on fasting blood glucose levels in non-diabetic Wistar rats.

Average Blood Glucose Level (mg/dL) at Different Time Interval and Percentage of Glycemia Changes Relative to Baseline Values					
Groups (n = 6)	0 min	30 min	60 min	120 min	180 min
Control (Tween-80, 2%)	149.25 ± 28.30	144.75 ± 13.09 (−3.01%)	146.75 ± 17.05 (−1.67%)	142.5 ± 24.33 (−4.52%)	146 ± 26.69 (−2.17%)
Metformin (100 mg/kg)	138.5 ± 18.69	117.25 ± 4.03 * (−15.34%)	106.75 ± 9.6 * (−22.92%)	106.5 ± 3.10 * (−23.10%)	112.5 ± 5.68 * (−18.77%)
*C. amoenum* (125 mg/kg)	145.25 ± 21.97	124.25 ± 13.93 * (−14.45%)	121.25 ± 14.97 * (−16.52%)	114.5 ± 13.17 * (−21.17%)	118.75 ± 11.67 * (−18.24%)
*C. amoenum* (250 mg/kg)	172.75 ± 37.28	136 ± 25.33 (−21.27%)	128.5 ± 18.69 (−25.61%)	115.5 ± 18.66 (−33.14%)	115.25 ± 14.38 (−33.28%)
*C. amoenum* (500 mg/kg)	169.75 ± 12.52	132 ± 22.77 (−22.23%)	124 ± 19.33 (−26.95%)	109 ± 15.76 (−35.78%)	114.25 ± 15.96 (−32.69%)

Data are expressed as mean ± standard deviation, * *p* < 0.05 compared with the control group at corresponding post-treatment time points.

**Table 6:** Effect of ethanolic extract on fasting blood glucose levels in glucose-loaded rats.

Average Blood Glucose Level (mg/dL) at Different Time Intervals (% of Glycemic Changes with Respect to Baseline Values)					
Groups (n = 6)	0 min	30 min	60 min	120 min	180 min
Control (Tween-80, 2%)	130 ± 14.44	172.75 ± 36.43 (32.88%)	139.5 ± 10.53 (7.30%)	125.5 ± 5.91 (−3.46%)	121 ± 5.59 (−6.92%)
Metformin (100 mg/kg)	142 ± 19.37	177.5 ± 19.87 (25%)	115.75 ± 14.05 (−18.48%)	116.75 ± 19.53 (−17.78%)	121 ± 16.75 (−14.78%)
*C. amoenum* (125 mg/kg)	138.75 ± 21.92	158.5 ± 4.2 (14.23%)	140 ± 1.41 (0.9%)	133.5 ± 7.72 (−3.78%)	134 ± 5.1 (−3.42%)
*C. amoenum* (250 mg/kg)	117.25 ± 20.61	151.25 ± 27.20 (28.99%)	131.5 ± 17.99 (12.15%)	125.25 ± 17.67 (6.82%)	130.75 ± 15.1 (11.51%)
*C. amoenum* (500 mg/kg)	147 ± 12.96	174.5 ± 22.66 (18.70%)	118.75 ± 11.64 (−19.21%)	120.25 ± 6.70 (−18.19%)	124 ± 5.03 (−15.64%)

Data are expressed as mean ± standard deviation.

**Table 7:** Molecular docking analysis of *C. amoenum* constituents against antidiabetic targets in terms of binding energies and active-site residue interactions.

Ligands	Targets	Top Docking Score (kcal/mol)	Active Site Residue
Lycorine	5U3A	−8.0	ASP197
Pratorinine		−8.2	ASP300
Palmatine		−7.7	GLU233
Lycorine	6GB1	−6.6	GLU26
Pratorinine		−7.0	GLY132
Palmatine		−6.4	Not specified

**Table 8:** In silico ADMET analysis of major constituents of the *C. amoenum* bulb extract.

Category	Parameter/Metric	Pratorinine	Lycorine	Palmatine
Drug-likeness	MW (≤500 Da)	265.07	287.12	352.15
	TPSA (0 ~ 140 Å^2^)	50.94	62.16	40.80
	Hydrogen Bond Acceptors (HBA ≤ 10)	4	5	5
	Hydrogen Bond Donors (HBD ≤ 5)	1	2	0
	Number of Rotatable Bonds (nRB)	1	0	4
	Lipinski’s Rule of 5 (LR5)	Accepted	Accepted	Accepted
Absorption	LogP (Optimal: 0 ~ 3)	2.288	−0.268	2.893
	LogS (Water Solubility)	−4.078	−2.767	−4.820
	HIA (Human Intestinal Absorption)	Category 1, <30%	Category 0, >30%	Category 0, >30%
	Caco-2 Permeability (log unit)	−4.822	−5.148	−5.042
Distribution	VD (Volume of Distribution)	0.485	1.314	0.955
Metabolism	CYP1A2 Inhibitor	Poor Inhibition	Good Inhibition	Good Inhibition
Elimination	CL (Clearance, mL/min/kg)	7.942	6.257	8.128
	T_1/2_ (Half-life, hours)	1.078	2.987	1.984
Acute Toxicity	Predicted LD_50_ (mg/kg)	1000 mg/kg	230 mg/kg	200 mg/kg
	Toxicity Class	Class 4 (Slightly Toxic)	Class 3 (Toxic)	Class 3 (Toxic)
Toxicity Endpoints	Hepatotoxicity (Pred/Prob)	Active/0.52	Inactive/0.87	Inactive/0.77
	Neurotoxicity (Pred/Prob)	Active/0.54	Inactive/0.61	Active/0.55
	Nephrotoxicity (Pred/Prob)	Inactive/0.52	Active/0.56	Inactive/0.63
	Respiratory Toxicity (Pred/Prob)	Active/0.61	Active/0.91	Active/0.81
	Cardiotoxicity (Pred/Prob)	Inactive/0.80	Inactive/0.75	Inactive/0.72
	Carcinogenicity (Pred/Prob)	Active/0.50	Active/0.60	Active/0.50
	Immunotoxicity (Pred/Prob)	Active/0.99	Active/0.87	Active/0.96
	Mutagenicity (Pred/Prob)	Active/0.63	Inactive/0.67	Active/0.57
	Cytotoxicity (Pred/Prob)	Inactive/0.69	Active/0.75	Active/0.56

## Data Availability

This article contains the original contributions made in this study. The corresponding authors can be contacted with any additional questions.

## Supplementary Materials

The following supporting information can be downloaded at https://www.mdpi.com/article/10.3390/antiox15081004/s1, Figure S1: Gallic acid standard calibration curve for phenolic content estimation; Figure S2: Quercetin standard calibration curve for total flavonoid content estimation. ARRIVE checklist. Ethics and Specimen Authentication.

## Abbreviations

The following abbreviations are used in this manuscript:

A_o_	Absorbance of the control
A_s_	Absorbance of the sample
Abs	Absorbance
ADMET	Absorption, Distribution, Metabolism, Excretion, and Toxicity
AMBER	Assisted Model Building with Energy Refinement
ANOVA	Analysis of variance
ARRIVE	Animal Research: Reporting of In Vivo Experiments
*C. amoenum*	*Crinum amoenum*
CL	Clearance
DNSA	3,5-Dinitrosalicylic acid
DPPH	2,2-Diphenyl-1-picrylhydrazyl
ESI	Electrospray ionization
FC	Folin–Ciocalteu
FRAP	Ferric reducing antioxidant power
GAFF	General AMBER Force Field
GAEs	Gallic acid equivalents
GI	Gastrointestinal
GLP-1	Glucagon-like peptide-1
GLP-1R	Glucagon-like peptide-1 receptor
HBA	Hydrogen bond acceptor
HBD	Hydrogen bond donor
HIA	Human intestinal absorption
HPLC	High-performance liquid chromatography
HSD	Honestly significant difference
IC_50_	Half-maximal inhibitory concentration
IUCN	International Union for Conservation of Nature
LC	Liquid chromatography
LC–ESI/MS	Liquid chromatography–electrospray ionization mass spectrometry
LC-MS	Liquid chromatography–mass spectrometry
LD_50_	Median lethal dose
LR5	Lipinski’s Rule of Five
MD	Molecular dynamics
m/z	Mass-to-charge ratio
MW	Molecular weight
NC3Rs	National Centre for the Replacement, Refinement and Reduction of Animals in Research
NIH	National Institutes of Health
NMR	Nuclear magnetic resonance
nRB	Number of rotatable bonds
NPT	Isothermal–isobaric ensemble
NVT	Canonical ensemble
OECD	Organisation for Economic Co-operation and Development
OGTT	Oral glucose tolerance test
PDB	Protein Data Bank
PDBQT	Protein Data Bank, Partial Charge, and Atom Type format
p.o.	Per os/oral administration
PURC	Pokhara University Research Center
QE	Quercetin equivalents
RoG	Radius of gyration
Rgyr	Radius of gyration
RMSD	Root mean square deviation
RMSF	Root mean square fluctuation
RT	Retention time
SNP	Sodium nitroprusside
T1_/2_	Half-life
TFC	Total flavonoid content
TLC	Thin-layer chromatography
TPC	Total phenolic content
TPSA	Topological polar surface area
UV	Ultraviolet
UV–Vis	Ultraviolet–visible
VD	Volume of distribution
WHO	World Health Organization
