# Supplementary material for: Antioxidant and Antidiabetic Activity of the Bulb Extract of Crinum amoenum Roxb. ex Ker Gawl: An Integrated In Vitro, In Vivo and In Silico Approach
Source: Antioxidants (Basel). 2026 Aug 13;15(8):1004. doi: 10.3390/antiox15081004 (PMC13509966; doi:10.3390/antiox15081004)
Supplement: Supplementary file 1 [file antioxidants-15-01004-s001.zip › ARRIVE checklist.pdf]

NOTE: Please save this file locally before filling in the table, DO NOT work on the file within your internet browser as changes will not be saved. Adobe Acrobat Reader (available free here) is recommended for completion.

The ARRIVE guidelines 2.0: author checklist

The ARRIVE Essential 10

These items are the basic minimum to include in a manuscript. Without this information, readers and reviewers cannot assess the reliability of the findings.

Item	#	Recommendation (abbreviated)	Section / line number, or reason for not reporting
**The ARRIVE Essential 10**			
**Study design**	1	a. Groups compared, including controls (rationale if no control).<br>b. Experimental unit.	Section 2.7.1 "Experimental Animals, Housing, and Humane Endpoint"; Section 2.7.2 "Acute Toxicity Study"; Section 2.7.3 "In Vivo Determination of Hypoglycemic Activity". Five groups: vehicle control (2% Tween), metformin 100 mg/kg, and extract 125, 250 and 500 mg/kg; distilled-water control in the acute toxicity study.<br>Experimental unit = the individual rat (Section 2.7.1, 2.7.3).
**Sample size**	2	a. Exact number per group and total number of animals.<br>b. How sample size was decided / a priori calculation.	a. Section 2.7.1: 35 Wistar rats in total — 30 males allocated to five groups (n = 6/group) for the hypoglycaemic and OGTT experiments, 5 females for the acute toxicity study. Also n=6/group in Section 2.7.3 and in Tables 5 and 6.<br>b. Section 2.7.1: stated explicitly that no formal power calculation was performed; group size based on comparable published pharmacological studies and on the 3Rs principle of reduction.
**Inclusion and exclusion criteria**	3	a. Criteria for including/excluding animals and data points; a priori or not.<br>b. Animals or data points not analysed, with reasons.<br>c. Exact n per group in each analysis.	a. Section 2.7.1: animals with signs of illness, abnormal behavior, injury, pregnancy or failure to acclimatise were excluded (criteria set before the experiment).<br>b. Section 2.7.1: no animals were excluded after group allocation; all 30 rats in the hypoglycemic/OGTT experiment were analyzed.<br>c. Section 2.7.1 and 2.7.3 (n = 6/group); footnotes of Tables 5 and 6 (Section 3.8.1, 3.8.2).
**Randomisation**	4	a. Whether randomization was used and how the sequence was generated.<br>b. Strategy to minimize confounders.	a. Section 2.7.1: animals allocated by a computer-generated randomization procedure after acclimatization; Section 2.7.2: the five female rats for acute toxicity were selected at random.<br>b. Section 2.7.1 (allocation after a two-week acclimatization, groups matched for baseline body weight) and Section 2.7.3 (one-week washout between the normoglycaemic and OGTT experiments to avoid carry-over; 18 h fast standardized before every measurement).
**Blinding**	5	Who was aware of group allocation at each stage (allocation, conduct, outcome assessment, analysis).	Section 2.7.1 states that investigators responsible for treatment administration and/or outcome assessment were blinded where applicable.<br>NOTE — please strengthen this sentence before submission (see "Points to strengthen", item 5). ARRIVE asks stage-by-stage who was and was not blinded.
**Outcome measures**	6	a. All outcome measures defined.<br>b. Primary outcome measure for hypothesis-testing studies.	a. Section 2.7.2 (behavioral signs, morbidity and mortality over 14 days) and Section 2.7.3 (blood glucose from tail-vein blood by calibrated glucometer at 0, 30, 60, 120 and 180 min; percentage change from the 0 min baseline). Results in Sections 3.7, 3.8.1 and 3.8.2.<br>b. Primary outcome = percentage reduction in blood glucose relative to baseline (Sections 2.7.3, 3.8.1,

			3.8.2). Sample size was not determined from it — see item 2b.
**Statistical methods**	**7**	a. Statistical methods for each analysis, including software.<br>b. Assessment of the assumptions of the statistical approach.	a. Section 2.9 "Statistical Analysis": mean ± SD; two-way ANOVA with Tukey HSD post hoc for the in vivo hypoglycemic data; IC50 from dose–response curves; IBM SPSS Statistics v19 and Microsoft Excel 2013; significance at $p < 0.05$.<br>b. Not currently reported — please add one sentence on normality/homogeneity of variance testing (see "Points to strengthen", item 7b).
**Experimental animals**	**8**	a. Species, strain, sex, age, weight.<br>b. Provenance, health status, genetic status, previous procedures.	a. Section 2.7.1: Wistar albino rats, 200–250 g, 8–10 weeks old; 30 males (hypoglycemic/OGTT) and 5 females (acute toxicity). Section 2.7.2: non-pregnant, nulliparous females.<br>b. Section 2.7.1: supplied by the Animal House Division, Department of Plant Resources, Kathmandu, Nepal; healthy, conventional (non-genetically modified) animals. Section 2.7.3 states that the same animals were reused after a one-week washout, i.e. previous procedure disclosed.
**Experimental procedures**	**9**	a. What was done, how, and with what.<br>b. When and how often.<br>c. Where, including acclimatization.<br>d. Why (rationale).	a. Sections 2.7.2 and 2.7.3: oral (p.o.) dosing of extract (125, 250, 500 mg/kg), metformin 100 mg/kg, 2% Tween vehicle, dextrose 2 g/kg; OECD 425 sequential dosing at 2000, 3000 and 5000 mg/kg; tail-vein sampling with a calibrated glucometer. Anesthesia (ketamine 87 mg/kg + xylazine 13 mg/kg, i.m.) and euthanasia in Section 2.7.1.<br>b. Section 2.7.2: observation continuously for 4 h at 30 min intervals, then daily for 14 days. Section 2.7.3: 18 h fast; glucose load 1 h after treatment; sampling at 0, 30, 60, 120 and 180 min.<br>c. Section 2.7.1: animal research facility, Pokhara University; polypropylene cages; two-week acclimatization.<br>d. Section 2.7.3 (dose and model rationale, washout to reduce animal use), Section 2.7.2 (OECD 425), and the final paragraph of the Introduction (study objectives).
**Results**	**10**	a. Descriptive statistics per group with a measure of variability.<br>b. Effect size with confidence interval, if applicable.	a. Section 3.7 (acute toxicity), Section 3.8.1 and Table 5 (non-diabetic rats), Section 3.8.2 and Table 6 (glucose-loaded rats): mean ± SD with n stated; in vitro data in Sections 3.3–3.6 (mean ± SD, n = 3).<br>b. Effect sizes are given as percentage change in blood glucose from baseline; confidence intervals are not reported. Please either add 95% CIs or state the reason (see "Points to strengthen", item 10b).
**The Recommended Set**			
**Abstract**	**11**	Objectives, species/strain/sex, key methods, main findings, conclusions.	Abstract (page 1). Objectives, methods (in vitro, in vivo in Wistar rats, in silico), principal findings and conclusion are given.<br>NOTE: the abstract states "Wistar rats" but not the sex of the animals — adding "male Wistar rats (and female rats for acute toxicity)" would fully satisfy item 11.
**Background**	**12**	a. Scientific background and rationale.<br>b. Why this species/model addresses the objectives and its relevance to human biology.	a. Introduction, paragraphs 1–5 (traditional use of *C. amoenum* in Nepal, phytochemistry of the genus Crinum, rationale for α-amylase and GLP-1R as targets, rationale for lycorine, pratorinine and palmatine).<br>b. Introduction (α-amylase inhibition as the accepted basis of the postprandial action of acarbose) and Discussion, Section 4 (rat OGTT as an accepted model of postprandial glycaemia; relevance to human pancreatic α-amylase, PDB 5U3A).
**Objectives**	**13**	Research question, objectives and hypotheses.	Final two paragraphs of the Introduction (Section 1), which list the screening, quantification, LC-MS annotation, in vitro, in vivo and in silico objectives and state explicitly what was outside the scope.
**Ethical statement**	**14**	Name of the ethical review body, licence/protocol	Section 2.1 "Ethics Statement": Institutional Review

		number.	Committee (IRC), Pokhara University Research Centre (PURC); Protocol No. Ref. No. 105-079/80; approval date 9 January 2023; NIH guidelines followed. Repeated in the "Institutional Review Board Statement" in the back matter. Approval letter supplied as supplementary documentation.
**Housing and husbandry**	**15**	Housing and husbandry conditions, including enrichment.	Section 2.7.1: polypropylene cages in the animal research facility of Pokhara University; natural 12 h light/dark cycle; 25 ± 3 °C; standard diet and water ad libitum; two-week acclimatization, per National Research Council recommendations [ref. 20].<br>NOTE: environmental enrichment is not described — state what was provided, or state that none was provided.
**Animal care and monitoring**	**16**	a. Steps to reduce pain, suffering and distress.<br>b. Adverse events.<br>c. Humane endpoints and monitoring frequency.	a. Section 2.1 and Section 2.7.1: all efforts made to minimize suffering (NIH/NC3Rs 3Rs principles); terminal deep anesthesia with ketamine 87 mg/kg + xylazine 13 mg/kg i.m. before euthanasia; reuse of animals after washout to reduce total numbers (Section 2.7.3).<br>b. Section 3.7: no behavioral alterations, no signs of illness and no mortality up to 5000 mg/kg; Section 2.7.2 lists the signs monitored (convulsions, writhing, diarrhea, weight loss, lethargy, tremors, paralysis, urination, food and water intake, morbidity, mortality).<br>c. Section 2.7.1 (heading) and Section 2.7.2 (monitoring for 4 h at 30 min intervals, then daily for 14 days). The specific humane endpoint criteria should be spelled out — see "Points to strengthen", item 16c.
**Interpretation / scientific implications**	**17**	a. Interpretation in the light of objectives and the literature.<br>b. Limitations, sources of bias and imprecision.	a. Section 4 "Discussion" (whole section) and Section 5 "Conclusions".<br>b. Section 4, penultimate paragraph: tentative LC-MS annotation without standards or NMR; alkaloids not quantified or isolated; crude extract and a single dose level; no chemically induced diabetic model; no repeated dosing, insulin, HbA1c or histology; 180 min follow-up only; docking/MD/ADMET are computational predictions and the GLP-1R result is not evidence of receptor activation. Repeated in Section 5
**Generalisability / translation**	**18**	How the findings generalize to other species or conditions, and relevance to human biology.	Section 4 "Discussion" (docking against human pancreatic α-amylase and human GLP-1R; predicted human intestinal absorption and half-life of 1–3 h discussed in relation to postprandial glucose control) and Section 5 "Conclusions" (pratorinine reported as an in silico lead only; bioassay-guided fractionation and testing of purified compounds identified as the necessary next step).
**Protocol registration**	**19**	Whether a protocol was prepared before the study and where it was registered.	Not currently stated. The study protocol was reviewed and approved in advance by the IRC of PURC (Section 2.1) but was not registered in a public preclinical registry. Please add the one-line statement given in "Points to strengthen", item 19.
**Data access**	**20**	Whether and where study data are available.	"Data Availability Statement" in the back matter: all original contributions are included in the article; further enquiries may be directed to the corresponding authors.
**Declaration of interests**	**21**	a. Conflicts of interest, financial and non-financial.<br>b. Funding sources with grant identifier and the role of the funder.	a. "Conflicts of Interest" statement in the back matter: the authors declare no conflicts of interest.<br>b. "Funding" statement in the back matter: Ongoing Research Funding Program, King Saud University, Riyadh, Saudi Arabia, grant number ORF-2026-376. Add a short clause stating that the funder had no role in the design, analysis or reporting of the study (see "Points to strengthen", item 21b).
