# Supplementary material for: Antioxidant and Antidiabetic Activity of the Bulb Extract of Crinum amoenum Roxb. ex Ker Gawl: An Integrated In Vitro, In Vivo and In Silico Approach
Source: Antioxidants (Basel). 2026 Aug 13;15(8):1004. doi: 10.3390/antiox15081004 (PMC13509966; doi:10.3390/antiox15081004)
Supplement: Supplementary file 1 [file antioxidants-15-01004-s001.zip › Ethics and Specimen Authentication.pdf]

Antioxidant and Antidiabetic Activity of *Crinum amoenum* Roxb. ex-Ker Gawl: Quantitative and Qualitative Analysis with an *In Silico* Approach

Ethical Approval Letter

पोखरा विश्वविद्यालय
POKHARA UNIVERSITY
Kaski, Nepal

चलानी नं.:/Ref.No. 105-079/80

Date: 9th Jan 2023

Mr./Ms. Prabhat Kumar Jha
Principal Investigator
School of Health and Allied Sciences
Faculty of Health Sciences
Pokhara University

Ref: Ethical Approval of Research Proposal

Dear Mr./Ms. Jha

It is our pleasure to inform you that Institutional Review Committee (IRC), Pokhara University has approved your proposal for the ethical approval as per the decision taken by the committee on dated **9th Jan 2023** Meeting number **60.**

Research title: ***"Evaluation of anti-inflammatory, analgesic and hypoglycemic activity of medicinally important plants of Nepal"***

As per IRC rules and regulations, the investigator has to strictly follow the protocol mentioned in the proposal. Any change in objective(s), problem statement, research question, hypothesis, methodology, implementation procedure, data management and budget that may be necessary in course of the implementation of the research proposal can only be made so and implemented after prior approval from this committee. Thus, it is compulsory to submit the detail of such changes intended or desired with justification prior to actual change in the protocol.

Further, the researchers are directed to strictly abide by the IRC during the implementation of their research proposal and submit progress report and full copy of summary report upon completion.

If you have any questions, please contact the IRC, Pokhara University.

Thank You

....
Associate Professor Dr. Niranjan Shrestha
Chairperson
Institutional Review Committee
Pokhara University
puirc@pu.edu.np, nrjanstha@gmail.com

CS CamScanner

Plant Herbarium Specimen Authentication Letter

नेपाल सरकार

वन तथा वातावरण मन्त्रालय

वनस्पति विभाग

विश्व शान्ति जैविक विविधता उद्यान

रानीवन, पोखरा

पत्र संख्याः 065/80

च. नं 99

मिति २०७९/०८/०८

जो जस संग सम्बन्धित छ ।

श्री प्रभात कुमार झाले लेखनाथ-३०, कास्की, नेपालबाट मिति २०७९/०३/०५ गते संकलन गरेको P२ संकलन नम्बर रहेको बनस्पतिको मृत नमुना (Herbarium)को वैज्ञानिक पहिचान गरिपाऊ भनि दिएको निबेदन उपर बिभिन्न श्रोतबाट अध्ययन गर्दा उक्त बनस्पतिको उपलब्ध भएका भागहरुलाइ सन्दर्भ सामग्रीकोरुपमा लिदा यो बनस्पतिक परिवार Amaryllidaceae भित्र पर्ने *Crinum amoenum* प्रजाति रहेको प्रमाणित गरिन्छ ।

क्र.स	बनस्पतिक परिवार	वैज्ञानिक नाम
१	Amaryllidaceae	*Crinum amoenum*

धर्मराज कोइराला

स.वै.अ

(२१६७८)
