# Supplementary material for: Antioxidant and Antidiabetic Activity of the Bulb Extract of Crinum amoenum Roxb. ex Ker Gawl: An Integrated In Vitro, In Vivo and In Silico Approach
Source: Antioxidants (Basel). 2026 Aug 13;15(8):1004. doi: 10.3390/antiox15081004 (PMC13509966; doi:10.3390/antiox15081004)
Supplement: Supplementary file 1 [file antioxidants-15-01004-s001.zip › Supplemental Figures S1 and S2.pdf]

Antioxidant and Antidiabetic Activity of the Bulb Extract of *Crinum amoenum* Roxb. ex Ker Gawl: An Integrated *In Vitro*, *In Vivo* and *In Silico* Approach

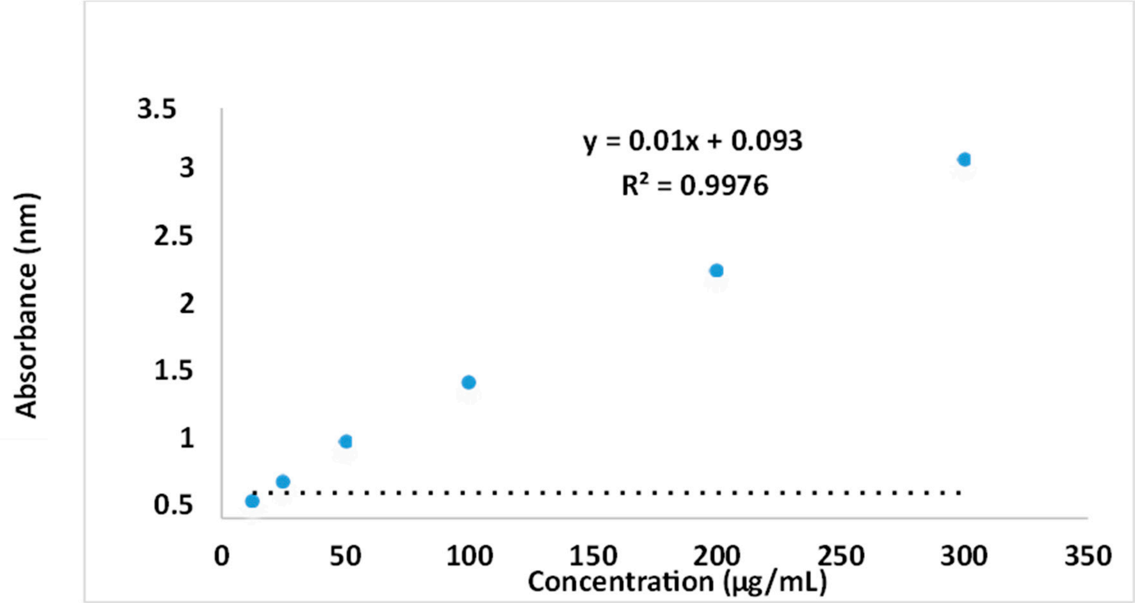

Figure S1. Gallic acid standard calibration curve for phenolic content estimation.

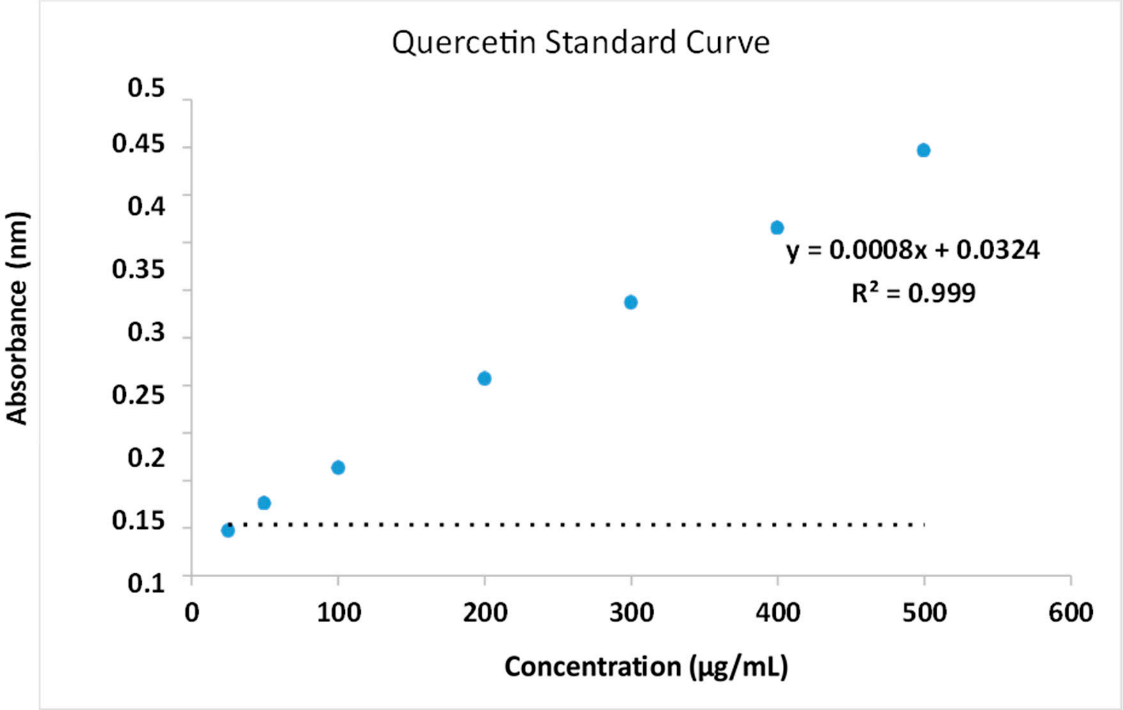

Figure S2. Quercetin standard calibration curve for total flavonoid content estimation.
